# Exploring the Association Between Mental Disorders and Various Arrhythmias via Mendelian Randomization Methods

**DOI:** 10.1111/gbb.70058

**Published:** 2026-06-18

**Authors:** Jingyang Su, Jialin Zhang, Yueyue Ding, Yongjun Ying

**Affiliations:** ^1^ Department of General Internal Medicine Tongde Hospital Affiliated to Zhejiang Chinese Medical University (Tongde Hospital of Zhejiang Province) Hangzhou China; ^2^ The Second Affiliated Hospital of Zhejiang Chinese Medical University (Xinhua Hospital of Zhejiang Province) Hangzhou China; ^3^ Department of Geriatrics Tongde Hospital Affiliated to Zhejiang Chinese Medical University (Tongde Hospital of Zhejiang Province) Hangzhou China

**Keywords:** arrhythmias, bipolar disorder, major depression, schizophrenia

## Abstract

Major depression, bipolar disorder, and schizophrenia are common mental illnesses, and their potential association with arrhythmias has long been a focus of clinical and research interest. To explore the possible causal relationship between these mental disorders and arrhythmias, we conducted a bidirectional two‐sample Mendelian randomization (MR) analysis using publicly available GWAS summary statistics. The primary analysis employed the inverse‐variance weighted (IVW) method, with sensitivity analyses including MR‐Egger regression and the weighted median estimator to assess robustness and address potential pleiotropy. Results indicated that genetic predisposition to major depression was associated with increased risks of atrial fibrillation/flutter (IVW: OR = 1.214, 95% CI: 1.092–1.349, *p* < 0.001), paroxysmal tachycardia (IVW: OR = 1.493, 95% CI: 1.261–1.769, *p* < 0.001), and atrioventricular block (IVW: OR = 1.257, 95% CI: 1.147–1.377, *p* < 0.001). Reverse MR also suggested a modest effect of atrioventricular block on depression risk (IVW: OR = 1.045, 95% CI: 1.011–1.079, *p* = 0.008). In conclusion, from the perspective of genetic liability and using the MR framework, our analysis supports a causal role of major depression in increasing the risk of several arrhythmias and suggests potential bidirectional causal effects between depression and atrioventricular block.

## Graphical abstract

Based on the validity of instrumental variable assumptions, our Mendelian randomization analysis revealed that genetic predisposition to major depression was associated with an increased risk of arrhythmias and suggested potential bidirectional effects between major depression and atrioventricular block.

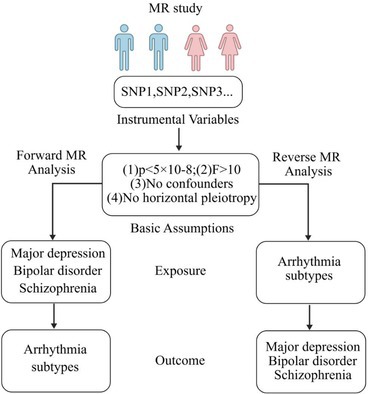

## 1 Introduction

Mental disorders have emerged as one of the fastest‐growing disease burdens globally. Epidemiological studies document a striking 48.1% increase in prevalence, with cases rising from 654.8 million in 1990 to 970.1 million in 2019 [1]. Major psychiatric conditions include major depression, bipolar disorder, and schizophrenia. Emerging evidence reveals substantial genetic and clinical overlaps between mood disorders, such as major depression and bipolar disorder, indicating potential shared disease mechanisms [2, 3, 4]. These disorders show strong correlations not only with suicidal behaviors but also with increased cardiovascular disease risks. Accounting for 40.5% of global disability‐adjusted life years, mental disorders impose severe burdens on individual wellbeing, family dynamics, and healthcare infrastructure [5]. The chronic nature, high recurrence rates, and frequent physical comorbidities associated with these conditions present significant challenges for public health systems worldwide.

Atrial fibrillation, the most common supraventricular tachyarrhythmia, represents a major clinical form of cardiac arrhythmia [6]. This condition arises from disorganized atrial electrical activity leading to compromised mechanical function. Current estimates indicate 2.2 million affected individuals in the United States and 4.5 million in the European Union experience either paroxysmal or persistent atrial fibrillation [7]. These cases constitute over 30% of all arrhythmia‐related hospital admissions and are strongly linked to heightened risks of stroke, heart failure, and mortality [8, 9]. Prevalence demonstrates a clear age‐related pattern, affecting 3%–5% of individuals over 60 years and reaching 8% among octogenarians [7]. Projections estimate the US patient population will exceed 5 million cases by 2050 [10].

Growing evidence supports a robust association between cardiac arrhythmias and psychiatric disorders. Atrial fibrillation patients show increased rates of comorbid depression (24.3%) and anxiety (14.5%), with particularly high prevalence in elderly populations (depression 40.3%, anxiety 33.6%) and Europe and North America [11]. Schizophrenia patients demonstrate higher incidence of prolonged QTc intervals (3.4% vs. 1.1%) and pathological Q waves (5.3% vs. 3.9%) compared to controls [12]. Psychological factors including anxiety, anger, depressive symptoms, and occupational stress independently contribute to atrial fibrillation risk [13]. A large cohort study of 6,576,582 participants identified mental disorders in 10% of subjects, with 8932 incident atrial fibrillation cases during follow‐up [14]. After multivariate adjustment, mental disorders maintained a significant association with increased atrial fibrillation risk. Specifically, bipolar disorder and schizophrenia patients showed a twofold higher atrial fibrillation risk, while depression, insomnia, or anxiety conferred a 1.5‐ to 1.7‐fold increased risk [14]. Targeted mental health interventions may reduce the global atrial fibrillation burden and associated healthcare costs, though large‐scale randomized trials are needed for definitive confirmation.

Given the practical limitations of clinical randomized controlled trials (RCTs), such as high costs and ethical constraints, Mendelian randomization (MR) has emerged as a powerful approach for causal inference in observational data. MR uses germline genetic variants as instrumental variables to mimic the random allocation of RCTs, thereby reducing confounding and reverse causality [15]. This method relies on three core assumptions: the genetic variants must be strongly associated with the exposure (relevance assumption), not associated with confounders (independence assumption), and affect the outcome only through the exposure (exclusion restriction assumption).

While previous MR studies have yielded inconsistent results regarding depression and atrial fibrillation risk, few have systematically examined multiple arrhythmia subtypes [16, 17, 18]. Our study extends this literature by comprehensively evaluating bidirectional causal relationships between several mental disorders and a range of arrhythmia subtypes—including atrial fibrillation/flutter, paroxysmal tachycardia, and atrioventricular block. It is important to note that MR estimates reflect the lifelong effect of genetically predicted exposure levels rather than the effects of short‐term interventions or behavioral changes. Despite its advantages, MR results may still be susceptible to horizontal pleiotropy. This study thus provides novel genetic insights into the psychocardiac axis, with an emphasis on subtype‐specific associations and robust bidirectional causal assessment.

## 2 Methods

### 2.1 Research Design

**FIGURE 1:**
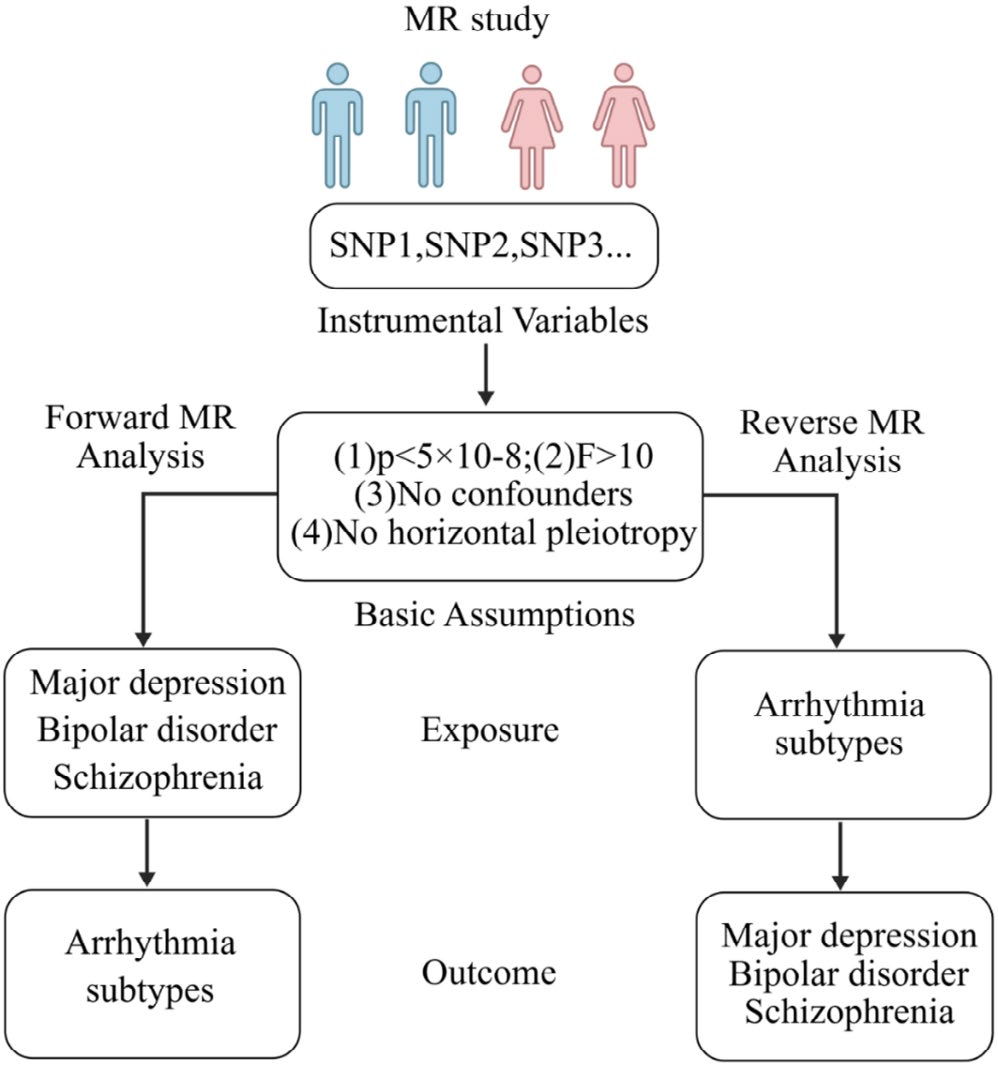
Flowchart of bidirectional Mendelian randomization.

We conducted a two‐sample Mendelian randomization (MR) study utilizing summary‐level data from genome‐wide association studies (GWAS) to investigate potential causal relationships between psychiatric disorders and cardiac arrhythmias, including their specific subtypes. This analytical approach relies on three fundamental assumptions (Figure 1): (1) The genetic variants (single nucleotide polymorphisms, SNPs) must exhibit robust associations with the exposures (psychiatric disorders or arrhythmias); (2) These SNPs must be independent of potential confounding factors (e.g., demographic characteristics, lifestyle factors); (3) The SNPs should influence the outcomes exclusively through the designated exposures (absence of horizontal pleiotropy). The study protocol conformed to STROBE‐MR guidelines [19], and all data were obtained from publicly available, de‐identified sources in accordance with the Helsinki Declaration and prevailing ethical standards.

### 2.2 Data Sources

Psychiatric disorder datasets were acquired from the Psychiatric Genomics Consortium (PGC): major depression (170,756 cases/329,443 controls) [20], schizophrenia (SCZ, 76,755 cases/243,649 controls) [21], and bipolar disorder (BD, 41,917 cases/371,549 controls) [22]. Arrhythmia phenotype data (including atrial fibrillation/flutter, paroxysmal tachycardia, and atrioventricular block) were derived from the FinnGen study (Release 12, https://r12.finngen.fi/). To mitigate population stratification bias, we restricted our analysis to individuals of European ancestry. Comprehensive dataset characteristics are presented in Table 1.

**TABLE 1:** Genetic data characteristics of mental disorders and arrhythmias.

Trait	Source	Dataset	Case (*n*)	Control (*n*)	Sample size	Population
Major depression	PGC	ieu‐b‐102	170,756	329,443	500,199	European
Bipolar disorder	PGC	ieu‐b‐5110	41,917	371,549	413,466	European
Schizophrenia	PGC	ieu‐b‐5099	76,755	243,649	320,404	European
Cardiac arrhythmias	FinnGen	finngen_R12_CARDIAC_ARRHYTM	92,926	288,216	381,142	European
Atrial fibrillation and flutter	FinnGen	finngen_R12_I9_AF	63,532	252,810	316,342	European
Paroxysmal tachycardia	FinnGen	finngen_R12_I9_PAROXTAC	12,878	252,810	265,688	European
Atrioventricular block	FinnGen	finngen_R12_I9_AVBLOCK	7850	375,343	383,193	European

### 2.3 Selection of Instrumental Variables

In forward MR analyses, psychiatric disorders served as exposures with arrhythmia phenotypes as outcomes, while reverse MR analyses inverted this relationship. Instrumental variable selection followed stringent criteria: genome‐wide significance (*p* < 5 × 10^−8^), linkage disequilibrium threshold (*r* ^2^ < 0.01 within 10,000 kb windows), and F‐statistic > 10 to ensure strong instruments [23, 24]. We systematically evaluated MR assumptions through: (1) Examination of SNP‐exposure associations (F‐statistics); (2) Assessment of potential confounding via GWAS catalog review (*p* > 0.05 for known confounders); (3) MR‐Egger regression testing for horizontal pleiotropy (intercept *p* > 0.05 considered acceptable).

### 2.4 Statistical Analysis

To ensure consistency in the allele direction of single nucleotide polymorphisms (SNPs) and to prevent bias from allele encoding discrepancies, the effect alleles of both exposure factors and outcomes were harmonized. Causal associations were evaluated using three complementary analytical methods:

1. *Inverse variance weighting (IVW)*: As the primary analytical method, IVW combines Wald ratio estimates from individual genetic variants using inverse‐variance weighting to provide an overall causal estimate. This approach assumes that all genetic variants satisfy the instrumental variable assumptions—specifically, no horizontal pleiotropy. In the absence of significant heterogeneity (Cochran's *Q* test *p* ≥ 0.05), a fixed‐effect IVW model was applied; otherwise, a random‐effects model was used. IVW offers high statistical power when the no‐pleiotropy assumption holds. Meanwhile, we used the false discovery rate (FDR) to correct the *p*‐values of IVW to improve the reliability of the data.
2. *MR‐egger regression*: MR‐Egger regression incorporates an intercept term that captures average directional pleiotropy—defined as the situation where genetic variants influence the outcome through pathways other than the exposure, and these pleiotropic effects exhibit a consistent directional bias. A statistically significant intercept (*p* < 0.05) suggests the presence of such pleiotropy, requiring causal estimates to be interpreted cautiously. MR‐Egger provides consistent causal estimates even when all genetic variants are invalid instruments, as long as the Instrument Strength Independent of Direct Effect (InSIDE) assumption is met. This method is particularly useful for detecting and correcting for pleiotropic bias [25].
3. *Weighted median estimator (WME)*: The WME method generates a consistent causal estimate if at least 50% of the weight in the analysis originates from valid instrumental variables. It is robust to invalid instruments and certain forms of pleiotropy, making it suitable for scenarios where a subset of genetic variants may violate MR assumptions. WME offers reliable inference when pleiotropy is present but not systematic in direction [26].

To assess heterogeneity, Cochran's *Q* test was applied to IVW estimates; significant heterogeneity (*p* < 0.05) prompted the use of random‐effects IVW and triangulation with WME results [26]. Furthermore, the influence of individual SNPs on overall results was visually inspected using forest plots, scatter plots, funnel plots, and leave‐one‐out sensitivity plots, all of which display effect estimates and corresponding 95% confidence intervals.

All analyses were performed in R (version 4.4.0) primarily using the “TwoSampleMR” package.

## 3 Results

### 3.1 Mendelian Randomization Analysis

Following rigorous quality control procedures, we acquired genetic datasets pertaining to mental disorders and arrhythmias. When mental disorders served as exposure factors, we merged the major depression dataset with the arrhythmia dataset, resulting in 49 SNPs available for analysis. Similarly, combining bipolar disorder and schizophrenia datasets with arrhythmia data yielded 49 and 153 SNPs, respectively. In the reverse direction, using arrhythmia as the exposure and mental disorders as outcomes, integration of the arrhythmia and mental illness datasets provided 68 SNPs. Merging atrial fibrillation and atrial flutter, paroxysmal tachycardia, and atrioventricular block datasets with mental disorder data yielded 104, 6, and 8 SNPs, respectively.

All retained SNPs exhibited F statistics greater than 10 (see Tables S1–S7), confirming their strength as instrumental variables.

### 3.2 Bidirectional Mendelian Randomization Results

We applied two‐sample bidirectional Mendelian randomization to examine causal relationships between mental disorders (major depression, bipolar disorder, and schizophrenia) and arrhythmias (arrhythmia, atrial fibrillation, atrial flutter, paroxysmal tachycardia, atrioventricular block).

#### 3.2.1 Forward MR: Mental Disorders as Exposures

**FIGURE 2:**
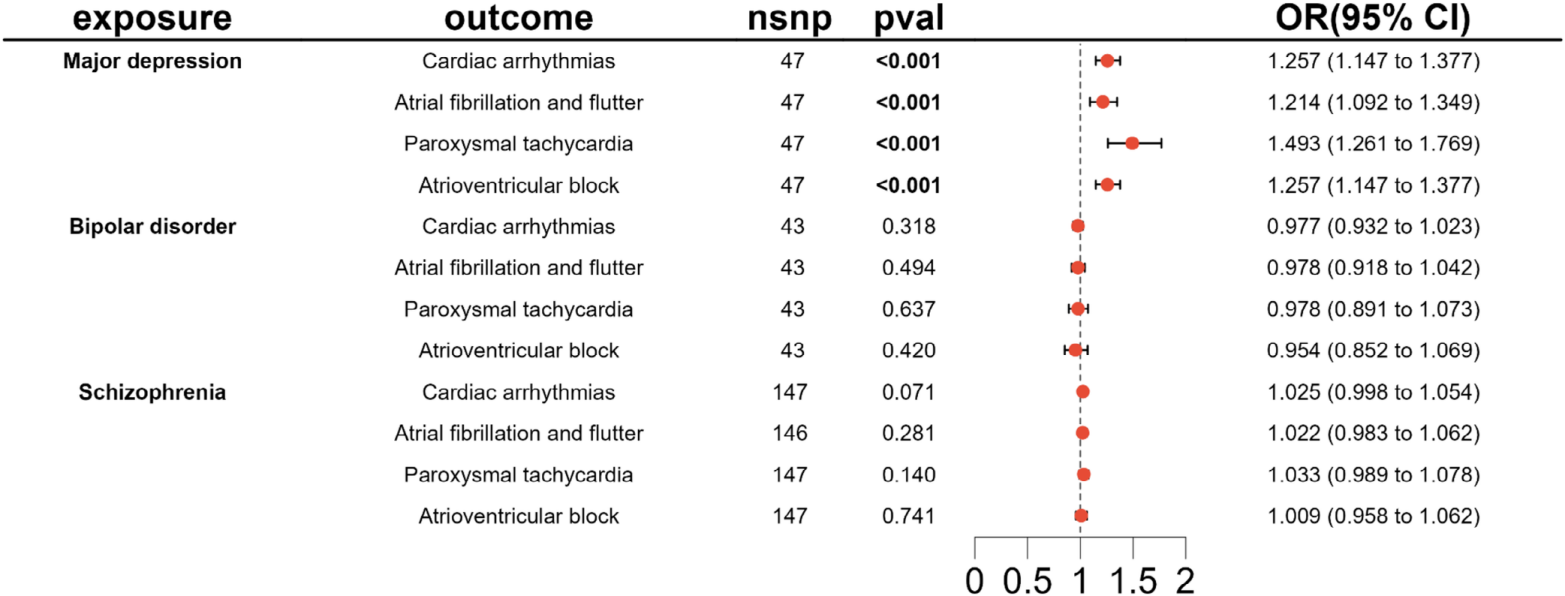
IVW results of mental disorders and arrhythmias.

Forward MR analysis indicated a significant causal effect of major depression on arrhythmia (summarized in Figure 2). The IVW method showed an odds ratio (OR) of 1.257 (95% CI: 1.147–1.377, *p* < 0.001), supported by the weighted median estimator (OR = 1.280, 95% CI: 1.152–1.422, *p* < 0.001). Major depression was also causally linked to atrial fibrillation and atrial flutter (IVW: OR = 1.214, 95% CI: 1.092–1.349, *p* < 0.001; WME: OR = 1.227, 95% CI: 1.069–1.408, *p* = 0.004), paroxysmal tachycardia (IVW: OR = 1.493, 95% CI: 1.261–1.769, *p* < 0.001; WME: OR = 1.375, 95% CI: 1.112–1.700, *p* = 0.003), and atrioventricular block (IVW: OR = 1.257, 95% CI: 1.147–1.377, *p* < 0.001; WME: OR = 1.280, 95% CI: 1.152–1.422, *p* < 0.001), as illustrated in Figure 2. No significant causal associations were observed for bipolar disorder or schizophrenia with any arrhythmia.

#### 3.2.2 Reverse MR: Arrhythmias as Exposures

**FIGURE 3:**
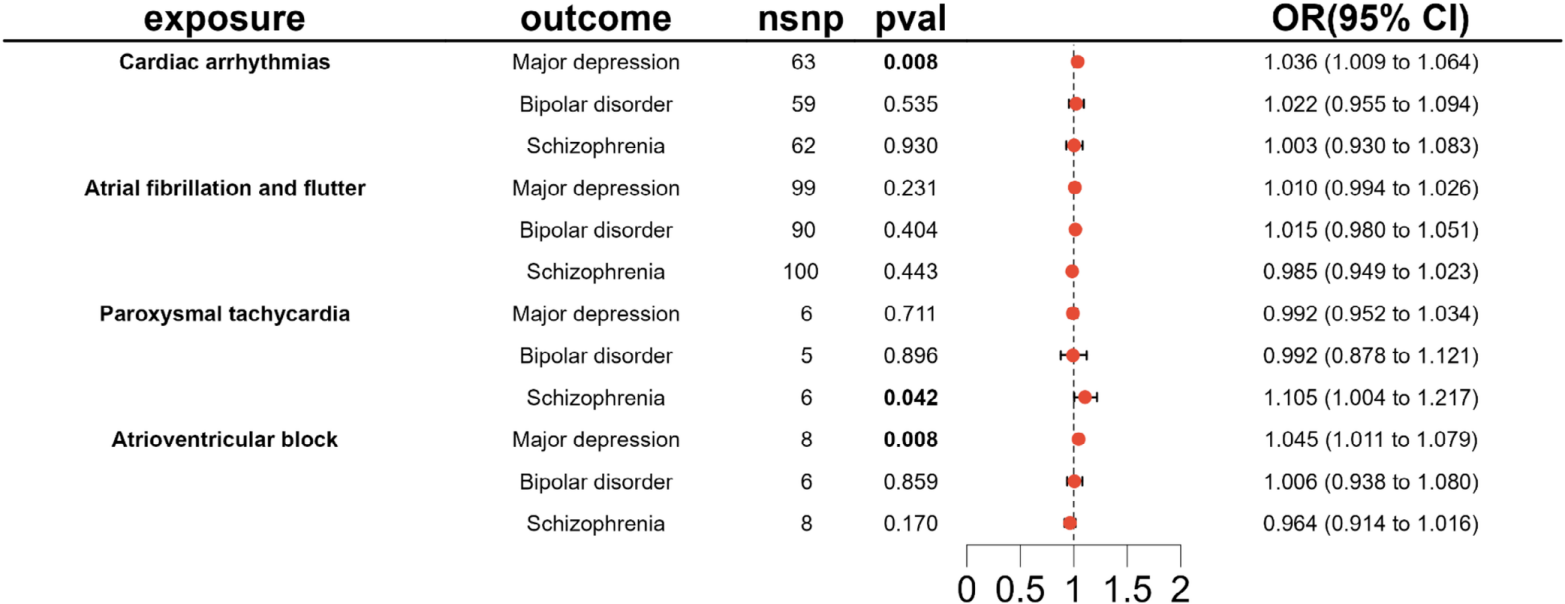
IVW results of arrhythmias and mental disorders.

Reverse MR analysis demonstrated a causal effect of arrhythmia on major depression (IVW: OR = 1.036, 95% CI: 1.009–1.064, *p* = 0.008; WME: OR = 1.046, 95% CI: 1.008–1.084, *p* = 0.016). Atrioventricular block was also associated with an increased risk of major depression (IVW: OR = 1.045, 95% CI: 1.011–1.079, *p* = 0.008). Paroxysmal tachycardia elevated the risk of schizophrenia (IVW: OR = 1.105, 95% CI: 1.004–1.217, *p* = 0.042; WME: OR = 1.106, 95% CI: 1.004–1.219, *p* = 0.042); however, there was no statistical difference in the results after FDR correction (Table 2). No causal relationship was identified between arrhythmia and bipolar disorder, as presented in Figure 3.

**TABLE 2:** Results of IVW and WME for mental disorders and arrhythmia.

Exposure	Outcome	IVW		WME	
		*p*	FDR(P)	OR (95% CI)	*p*
Major depression	Cardiac arrhythmias	0.000	0.000	1.280 (1.152–1.422)	0.000
	Atrial fibrillation and flutter	0.000	0.000	1.227 (1.069–1.408)	0.004
	Paroxysmal tachycardia	0.000	0.000	1.375 (1.112–1.700)	0.003
	Atrioventricular block	0.000	0.000	1.280 (1.152–1.424)	0.000
Bipolar disorder	Cardiac arrhythmias	0.318	0.637	0.955 (0.910–1.003)	0.067
	Atrial fibrillation and flutter	0.494	0.637	0.977 (0.911–1.048)	0.516
	Paroxysmal tachycardia	0.637	0.637	1.038 (0.929–1.160)	0.509
	Atrioventricular block	0.420	0.637	0.901(0.789–1.027)	0.119
Schizophrenia	Cardiac arrhythmias	0.071	0.280	1.008 (0.979–1.038)	0.602
	Atrial fibrillation and flutter	0.281	0.280	1.006 (0.965–1.048)	0.788
	Paroxysmal tachycardia	0.140	0.375	1.028 (0.970–1.089)	0.358
	Atrioventricular block	0.741	0.741	0.994 (0.926–1.068)	0.877
Cardiac arrhythmias	Major depression	0.008	0.024	1.046 (1.008–1.084)	0.016
	Bipolar disorder	0.535	0.803	0.989 (0.913–1.072)	0.796
	Schizophrenia	0.930	0.930	0.984 (0.911–1.062)	0.672
Atrial fibrillation and flutter	Major depression	0.231	0.443	1.012 (0.988–1.038)	0.323
	Bipolar disorder	0.404	0.443	0.977 (0.932–1.023)	0.322
	Schizophrenia	0.443	0.443	0.980 (0.944–1.018)	0.305
Paroxysmal tachycardia	Major depression	0.711	0.896	0.985 (0.935–1.037)	0.558
	Bipolar disorder	0.896	0.896	1.037 (0.909–1.184)	0.586
	Schizophrenia	0.042	0.126	1.106 (1.004–1.219)	0.042
Atrioventricular block	Major depression	0.008	0.024	1.032 (0.992–1.074)	0.121
	Bipolar disorder	0.859	0.225	1.006 (0.913–1.108)	0.907
	Schizophrenia	0.170	0.859	0.952 (0.891–1.018)	0.149

### 3.3 Sensitivity Analysis

We performed comprehensive sensitivity analyses to evaluate the robustness of our findings. Heterogeneity was assessed using Cochran's *Q* test for IVW and MR‐Egger regression (Table 3). A *p*‐value greater than 0.05 indicated absence of significant heterogeneity; where *p* was less than 0.05, a random‐effects model was applied to IVW analyses to improve reliability. Weighted median estimates were included as Supporting Information (Table 2).

**TABLE 3:** Horizontal pleiotropy in mental disorders and arrhythmias.

Exposure	Outcome	Heterogeneity test				Pleiotropy test		
		IVW		MR‐Egger		MR‐Egger		
		*Q*	*Q*_pval	*Q*	*Q*_pval	Intercept	Se	*p*
Major depression	Cardiac arrhythmias	83.716	0.001	83.714	0.000	0.000	0.008	0.975
	Atrial fibrillation and flutter	58.009	0.110	57.796	0.096	−0.004	0.010	0.686
	Paroxysmal tachycardia	63.114	0.048	62.750	0.041	0.008	0.015	0.612
	Atrioventricular block	83.716	0.001	83.714	0.000	0.000	0.008	0.975
Bipolar disorder	Cardiac arrhythmias	91.059	0.000	90.386	0.000	0.004	0.008	0.584
	Atrial fibrillation and flutter	87.272	0.000	87.062	0.000	0.003	0.011	0.755
	Paroxysmal tachycardia	79.569	0.000	78.409	0.000	−0.013	0.016	0.440
	Atrioventricular block	75.301	0.001	73.732	0.001	−0.018	0.020	0.356
Schizophrenia	Cardiac arrhythmias	354.718	0.000	352.535	0.000	0.004	0.004	0.345
	Atrial fibrillation and flutter	373.507	0.000	371.796	0.000	0.004	0.005	0.417
	Paroxysmal tachycardia	192.764	0.006	192.711	0.005	−0.001	0.006	0.843
	Atrioventricular block	174.814	0.052	174.483	0.048	0.004	0.007	0.601
Cardiac arrhythmias	Major depression	79.442	0.067	75.804	0.096	0.004	0.002	0.092
	Bipolar disorder	96.033	0.001	95.877	0.001	0.002	0.006	0.762
	Schizophrenia	201.656	0.000	201.383	0.000	−0.002	0.007	0.777
Atrial fibrillation and flutter	Major depression	162.542	0.000	162.462	0.000	0.000	0.002	0.828
	Bipolar disorder	141.606	0.000	139.050	0.000	0.004	0.004	0.207
	Schizophrenia	309.354	0.000	309.154	0.000	−0.001	0.004	0.801
Paroxysmal tachycardia	Major depression	2.404	0.791	1.597	0.809	−0.010	0.011	0.420
	Bipolar disorder	5.856	0.210	5.103	0.164	−0.021	0.032	0.553
	Schizophrenia	9.248	0.100	8.258	0.083	−0.018	0.027	0.527
Atrioventricular block	Major depression	8.783	0.269	6.884	0.332	0.012	0.009	0.246
	Bipolar disorder	4.482	0.482	3.917	0.417	0.021	0.028	0.494
	Schizophrenia	3.617	0.823	3.480	0.747	−0.006	0.016	0.724

Leave‐one‐out sensitivity analyses and funnel plots were generated to examine potential bias. Symmetrical funnel plots suggested minimal risk of publication bias. Leave‐one‐out analyses confirmed that no individual SNP disproportionately influenced the overall estimates, supporting result stability. Scatter plots visualized SNP effect relationships, and forest plots displayed Wald ratio estimates for individual SNPs. The MR‐Egger intercept test revealed intercepts near zero (all *p* > 0.05), indicating no substantial horizontal pleiotropy. Leave‐one‐out plots, funnel plots, scatter plots, and forest plots are available in the Supporting Information (Figures S1–S24).

## 4 Discussion

Through two‐way Mendelian randomization analysis, this study identified potential genetic causal associations between major depression and various arrhythmias. Forward Mendelian randomization (MR) analysis indicated that the genetic susceptibility to major depression significantly elevated the risks of atrial fibrillation and atrial flutter (IVW: OR = 1.214), paroxysmal tachycardia (OR = 1.493), and atrioventricular block (OR = 1.257), and the results of weighted median estimation were consistent. Reverse MR analysis suggested a reverse causal effect of arrhythmias on major depression (OR = 1.036). Among them, the genetic susceptibility to atrioventricular block mainly increased the risk of major depression (IVW OR = 1.045), and no significant association was found between bipolar disorder or schizophrenia and various arrhythmia. Mendelian randomization simulates a natural randomized experiment through genetic instrumental variables. Its core assumptions include a strong association, independence, and exclusion restrictions between the instrumental variables and the exposure results factors. The findings of this study reflect the long‐term effects of genetic susceptibility on the outcomes, rather than the short‐term phenotypic impacts that can be altered by clinical interventions. Compared with previous studies, this study has expanded both in depth and breadth. Most previous Mendelian randomization studies mainly focused on the association between the overall arrhythmia risk and mental disorders or were limited to common types such as atrial fibrillation. In contrast, this study is the first to systematically reveal the bidirectional genetic causal associations between MAJOR DEPRESSION and multiple specific arrhythmia subtypes (such as paroxysmal tachycardia and atrioventricular block). This finding not only verifies the overall connection between cardiac and mental health but also further details the genetic susceptibility characteristics of different types of arrhythmias, providing more precise targets for subsequent mechanistic research.

Methodologically, sensitivity analysis showed that there was heterogeneity in some results (Cochran's *Q p* < 0.05). We reduced its influence through a random‐effects model and multiple‐test correction (such as FDR). MR‐Egger regression did not find evidence of directional pleiotropy (intercept *p* > 0.05), and the results of weighted median estimation were consistent with those of IVW, supporting the robustness of the results. It is worth noting that the observed heterogeneity may stem from multiple aspects: first, the detected heterogeneity may be related to the genetic and environmental diversity within the European‐ancestry sub‐populations included in the constituent studies. Due to the geographical diversity of the European samples used, genetic testing tools may show different effect sizes in different European geographical regions; second, gene pleiotropy may affect outcomes through the nonexposure‐related pathways; third, the potential pathophysiological mechanisms of different arrhythmia subtypes are different, leading to the heterogeneous expression of the genetic structure. Although we have tried to control the influence of these factors through various sensitivity analyses, residual confounding is still difficult to completely eliminate.

Multiple pathophysiological mechanisms may underlie the increased risk of arrhythmias in individuals with mental disorders. A frequently proposed mechanism involves cardiac autonomic dysfunction, characterized by sympathetic predominance and parasympathetic withdrawal [27]. Experimental and clinical studies support this link: in animal models, stress‐induced depression led to increased heart rates, reduced heart rate variability (HRV), and exacerbated ventricular arrhythmias [28]. In humans with myocardial infarction, depression has been associated with reduced HRV, which partially mediates the relationship between depression and mortality [26]. Emotional factors may also influence arrhythmia risk by affecting cardiac repolarization stability. For instance, anger provocation in implantable cardioverter‐defibrillator (ICD) patients can induce T‐wave alternans and predict ventricular tachycardia/ventricular fibrillation events [29, 30]. Similarly, major depression has been linked to higher QT variability, a marker of arrhythmic susceptibility. Sex‐specific differences have been observed, with QT prolongation more prominent in women after acute coronary syndromes [31]. Central nervous system mechanisms might also be involved. The “brain‐heart lateralization hypothesis” suggests that lateralized emotional processing in the brain may correspond to asymmetric autonomic input to the heart, leading to repolarization abnormalities and potentially sudden cardiac death [27, 32]. Neuroimaging studies have shown associations between right midbrain activity and repolarization anomalies during psychological stress [32].

Additional pathways involve oxidative stress and neurohormonal activation [7, 33]. Mental disorders are associated with increased reactive oxygen species (ROS), which can alter the function of cardiac ion channels such as Nav1.5, encoded by SCN5A [34]. ROS‐mediated activation of transcription factors like NF‐κB may suppress SCN5A expression, reducing sodium current (INa) and increasing arrhythmia susceptibility [35, 36, 37]. Furthermore, metabolic disturbances such as elevated NADH levels, seen in conditions like Brugada syndrome, can promote mitochondrial ROS production and further disrupt ion channel function [7]. In atrial fibrillation (AF), autonomic influences may differ: parasympathetic activation often plays a more prominent role than in ventricular arrhythmias [38, 39]. Mental disorders may also exacerbate AF risk through hypothalamic–pituitary–adrenal axis dysregulation, renin‐angiotensin‐aldosterone system activation, serotonin signaling abnormalities, sleep disruption, and genetic factors [33].

Arrhythmias can increase the risk of mental disorders, particularly depression, through multiple mechanisms, reflecting a complex heart‐brain interaction [40]. The core pathophysiological mechanism involves dysfunction of the autonomic nervous system (ANS) [41]. Arrhythmias, especially bradyarrhythmias such as atrioventricular block, can lead to insufficient cerebral blood perfusion, causing cerebral ischemia and hypoxia, which directly impairs the function of neural circuits regulating mood [40]. Concurrently, abnormal cardiac rhythms act as a persistent physiological stressor that may activate the hypothalamic–pituitary–adrenal axis and the sympathetic nervous system via the heart‐brain axis [40], resulting in elevated levels of stress hormones such as cortisol and the release of inflammatory factors, collectively contributing to the development of depression [42, 43, 44]. This ANS imbalance, manifested as reduced heart rate variability, is both a feature of arrhythmia and a physiological marker of depression, forming a vicious cycle [45, 46, 47]. Furthermore, psychosocial factors cannot be overlooked; patients' concerns about their disease, reduced exercise tolerance, and fear of sudden death can exacerbate anxiety and depressive symptoms [48], while negative emotions may further aggravate ANS dysfunction. It is particularly noteworthy that certain medications used to treat arrhythmias (such as beta‐blockers) may also induce or worsen depressive symptoms through pharmacological effects [49]. Therefore, the comorbidity of arrhythmia and mental disorders is the outcome of intertwined physiological, neuroendocrine, and psychological factors.

The prevention and treatment of mental disorders require a comprehensive integrated approach that combines pharmacological and non‐pharmacological interventions. In terms of drug therapy, caution is paramount. Although antidepressants offer therapeutic benefits, some medications (such as tricyclic antidepressants and certain SSRIs/SNRIs) may carry risks of prolonging the QT interval, impairing cardiac conduction, or reducing vagal tone [49, 50, 51]. Hence, drug selection must involve a careful risk–benefit assessment, prioritizing agents with minimal cardiac impact, accompanied by close electrocardiographic monitoring. Non‐pharmacological interventions represent safe and effective core strategies. Psychotherapy (e.g., cognitive behavioral therapy) and interoception‐based interventions can help patients manage emotions and alleviate disease‐related distress [52]. Cardiac rehabilitation and aerobic exercise have been shown to simultaneously improve cardiovascular health and psychological state, though exercise prescriptions should be tailored to individual patient conditions [53, 54, 55]. More importantly, integrating psychological care into routine clinical practice is essential. This includes patient education about the disease and the provision of emotional support, thereby enhancing treatment adherence, improving clinical outcomes, and elevating quality of life [40, 53]. The American Heart Association (AHA) guidelines also recommend routine screening and management of depressive symptoms in patients with heart disease [53]. Ultimately, establishing a multidisciplinary collaborative model that integrates cardiology, psychiatry, and psychological services is the optimal pathway for managing such comorbid patients and breaking the vicious cycle of heart‐brain interplay.

This is the first study to date to systematically analyze the genetic associations between three major mental illnesses and arrhythmias using the bidirectional Mendelian randomization method. The Mendelian randomization study we conducted provides valuable insights into the possible bidirectional genetic relationship between mental disorders and arrhythmias, and also points out several important methodological considerations. Genetic factors may simultaneously influence the susceptibility to major depression and specific arrhythmias, and there is genetic evidence of a bidirectional causal association between the two. However, these results cannot be directly translated into clinical practice. To address the potential sample overlap issue in exposure and outcome data from genome‐wide association studies, we specifically selected datasets from different research consortia to minimize this concern. Nevertheless, we acknowledge that it is impossible to completely eliminate sample overlap, and any residual overlap may introduce bias in observational estimates.

There are some limitations that require our careful consideration. First, despite our efforts to minimize sample overlap, potential population stratification and the winner's curse in the single nucleotide polymorphism (SNP) selection process may still affect our study results. Although we employed robust sensitivity analyses including the MR‐Egger and weighted median methods, we recognize that we did not use methods such as CAUSE, MR‐PRESSO, or Steiger filtering to provide validation and improve the accuracy of our results. Second, the genetic susceptibility to depression is likely associated with some related traits (such as anxiety, sleep disorders, and drug use), which may independently affect the risk of arrhythmias. This makes it possible that our findings reflect common genetic influences or indirect effects rather than the direct causal effect of depression itself. Although our sensitivity analysis mainly focused on directional pleiotropy, we acknowledge that more complex pleiotropic pathways need to be further investigated through mediation analysis, which is also the focus of our future research. Third, the relatively weak effects of certain genetic instruments may limit statistical power, and the predominantly European ancestry of the available datasets restricts the generalizability of our findings and prevents their extension to other populations. Despite these limitations, molecular genetic analysis has obvious advantages over traditional observational studies as it can reduce confounding factors and reverse causality. This approach can effectively explore potential gene associations while overcoming the practical limitations and ethical concerns of long‐term follow‐up. Future studies should be extended to different populations, study subtypes of mental disorders, and combine clinical and experimental methods to clarify the underlying mechanisms.

## 5 Conclusion

We conducted a bidirectional Mendelian randomization study to explore potential genetic associations between major depression, bipolar disorder, schizophrenia, and various arrhythmia subtypes. Our findings suggest that genetic predisposition to depression is associated with an elevated risk of atrial fibrillation/flutter, paroxysmal tachycardia, and atrioventricular block. Conversely, genetic liability to atrioventricular block also showed a modest association with depression risk. While these results are consistent with a potential causal link, they reflect lifelong genetically predicted effects rather than short‐term clinical outcomes.

Potential biological pathways, such as autonomic nervous system dysregulation and oxidative stress, have been proposed in previous literature as plausible mechanisms underlying the mental health–arrhythmia relationship. However, these mechanisms were not directly tested in the present study and should be interpreted as hypothetical directions for future research. Further experimental and clinical studies are needed to validate these associations and elucidate the underlying pathophysiology.

## Funding

The authors have nothing to report.

## Ethics Statement

The authors have nothing to report.

## Consent

The authors have nothing to report.

## Conflicts of Interest

The authors declare no conflicts of interest.

## Supporting information


**Figure S1:** The causal effect of Major depression on Cardiac arrhythmias.
**Figure S2:** The causal effect of Major depression on Atrial fibrillation and flutter.
**Figure S3:** The causal effect of Major depression on Paroxysmal tachycardia.
**Figure S4:** The causal effect of Major depression on Atrioventricular block.
**Figure S5:** The causal effect of Bipolar disorder on Cardiac arrhythmias.
**Figure S6:** The causal effect of Bipolar disorder on Atrial fibrillation and flutter.
**Figure S7:** The causal effect of Bipolar disorder on Paroxysmal tachycardia.
**Figure S8:** The causal effect of Bipolar disorder on Atrioventricular block.
**Figure S9:** The causal effect of Schizophrenia on Cardiac arrhythmias.
**Figure S10:** The causal effect of Schizophrenia on Atrial fibrillation and flutter.
**Figure S11:** The causal effect of Schizophrenia on Paroxysmal tachycardia.
**Figure S12:** The causal effect of Schizophrenia on Atrioventricular block.
**Figure S13:** The causal effect of Cardiac arrhythmias on Major depression.
**Figure S14:** The causal effect of Cardiac arrhythmias on Bipolar disorder.
**Figure S15:** The causal effect of Cardiac arrhythmias on Schizophrenia.
**Figure S16:** The causal effect of Atrial fibrillation and flutter on Major depression.
**Figure S17:** The causal effect of Atrial fibrillation and flutter on Bipolar disorder.
**Figure S18:** The causal effect of Atrial fibrillation and flutter on Schizophrenia.
**Figure S19:** The causal effect of Paroxysmal tachycardia on Major depression.
**Figure S20:** The causal effect of Paroxysmal tachycardia on Bipolar disorder.
**Figure S21:** The causal effect of Paroxysmal tachycardia on Schizophrenia.
**Figure S22:** The causal effect of Atrioventricular block on Major depression.
**Figure S23:** The causal effect of Atrioventricular block on Bipolar disorder.
**Figure S24:** The causal effect of Atrioventricular block on Schizophrenia.
**Table S1:** 50 SNPs associated with depression.
**Table S2:** 52 SNPs associated with bipolar disorder.
**Table S3:** 154 SNPs associated with schizophrenia.
**Table S4:** 85 SNPs associated with cardiac arrhythmias.
**Table S5:** 128 SNPs associated with atrial fibrillation and flutter.
**Table S6:** 6 SNPs associated with paroxysmal tachycardia.
**Table S7:** 8 SNPs associated with paroxysmal tachycardia.

## Data Availability

The dataset presented herein can be accessed via an online database, whose name is provided in the article. The original contributions presented in the study are included in the article/Supporting Information, further inquiries can be directed to the corresponding authors.
