# Supplementary material for: Exploring the Association Between Mental Disorders and Various Arrhythmias via Mendelian Randomization Methods
Source: Genes Brain Behav. 2026 Jun 18;25(3):e70058. doi: 10.1111/gbb.70058 (PMC13276773; doi:10.1111/gbb.70058)
Supplement: Supplementary file 1 — Figure S1: The causal effect of Major depression on Cardiac arrhythmias. Figure S2: The causal effect of Major depression on Atrial fibrillation and flutter. Figure S3: The causal effect of Major depression on Paroxysmal tachycardia. Figure S4: The causal effect of Major depression on Atrioventricular block. Figure S5: The causal effect of Bipolar disorder on Cardiac arrhythmias. Figure S6: The causal effect of Bipolar disorder on Atrial fibrillation and flutter. Figure S7: The causal effect of Bipolar disorder on Paroxysmal tachycardia. Figure S8: The causal effect of Bipolar disorder on Atrioventricular block. Figure S9: The causal effect of Schizophrenia on Cardiac arrhythmias. Figure S10: The causal effect of Schizophrenia on Atrial fibrillation and flutter. Figure S11: The causal effect of Schizophrenia on Paroxysmal tachycardia. Figure S12: The causal effect of Schizophrenia on Atrioventricular block. Figure S13: The causal effect of Cardiac arrhythmias on Major depression. Figure S14: The causal effect of Cardiac arrhythmias on Bipolar disorder. Figure S15: The causal effect of Cardiac arrhythmias on Schizophrenia. Figure S16: The causal effect of Atrial fibrillation and flutter on Major depression. Figure S17: The causal effect of Atrial fibrillation and flutter on Bipolar disorder. Figure S18: The causal effect of Atrial fibrillation and flutter on Schizophrenia. Figure S19: The causal effect of Paroxysmal tachycardia on Major depression. Figure S20: The causal effect of Paroxysmal tachycardia on Bipolar disorder. Figure S21: The causal effect of Paroxysmal tachycardia on Schizophrenia. Figure S22: The causal effect of Atrioventricular block on Major depression. Figure S23: The causal effect of Atrioventricular block on Bipolar disorder. Figure S24: The causal effect of Atrioventricular block on Schizophrenia. Table S1: 50 SNPs associated with depression. Table S2: 52 SNPs associated with bipolar disorder. Table S3: 154 SNPs associated with schizophrenia. T [file GBB-25-e70058-s001.docx]

**
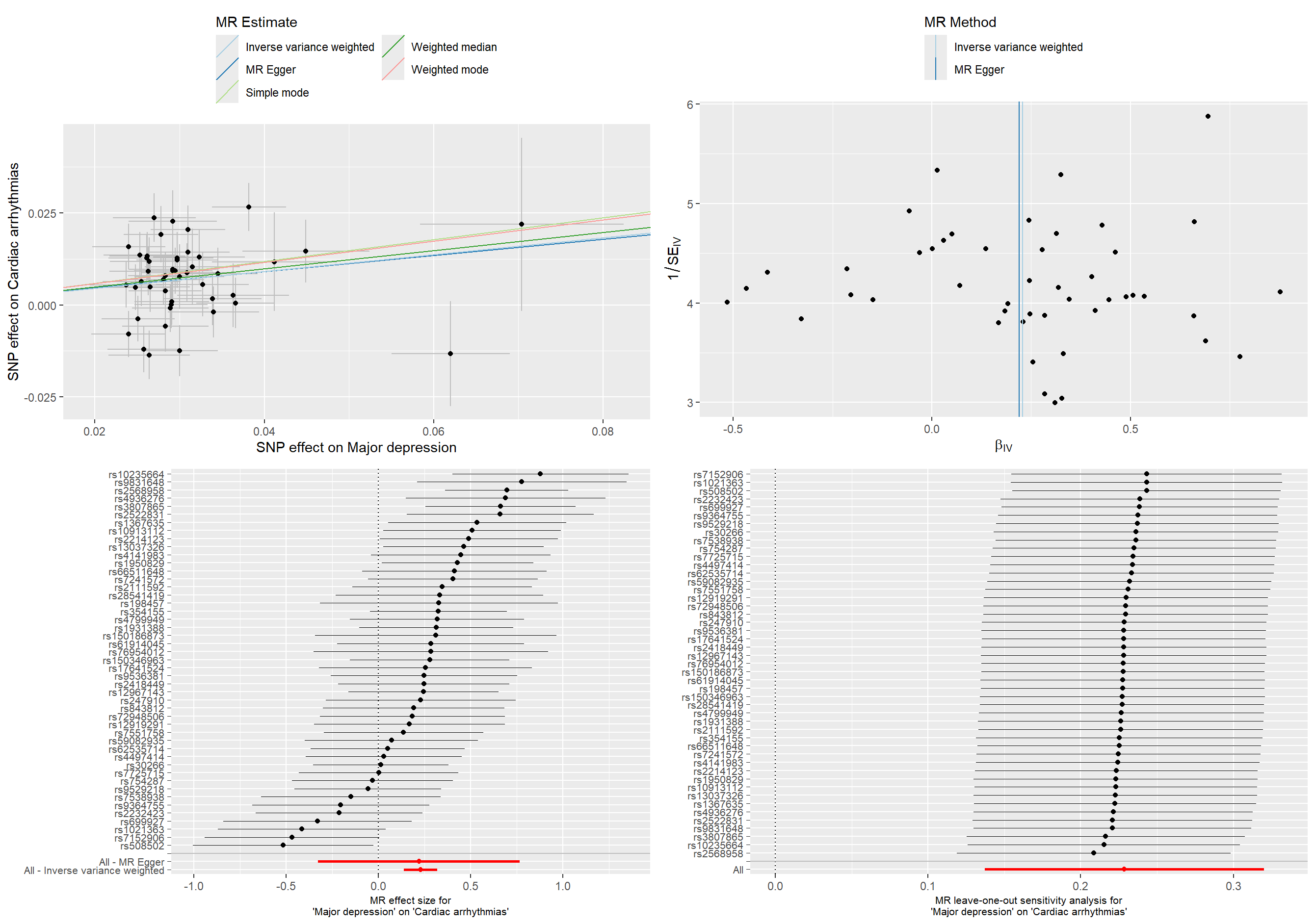
**

**Figure S1: The causal effect of** **Major depression on Cardiac arrhythmias**

**
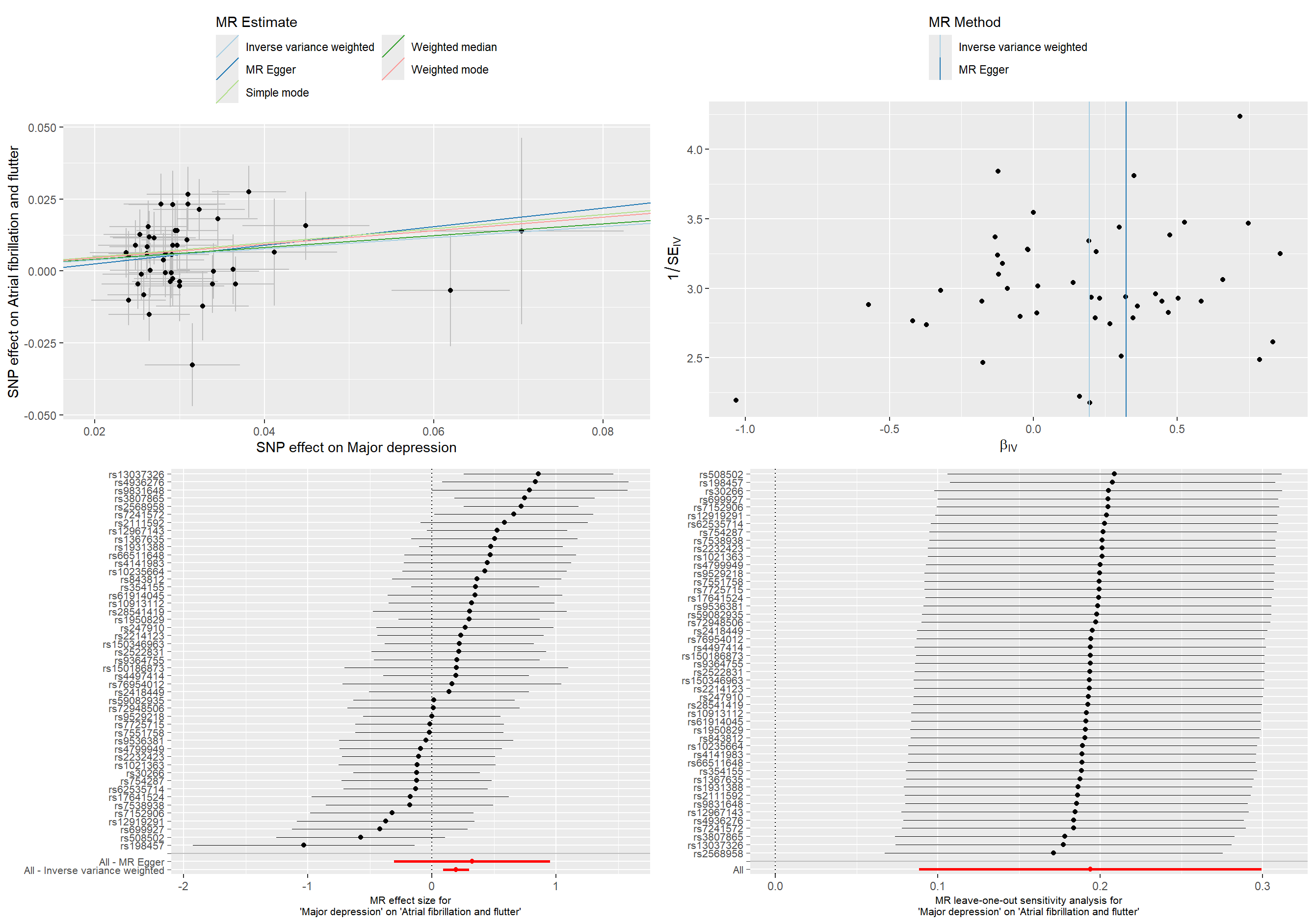
**

**Figure S2: The causal effect of** **Major depression on Atrial fibrillation and flutter**

**
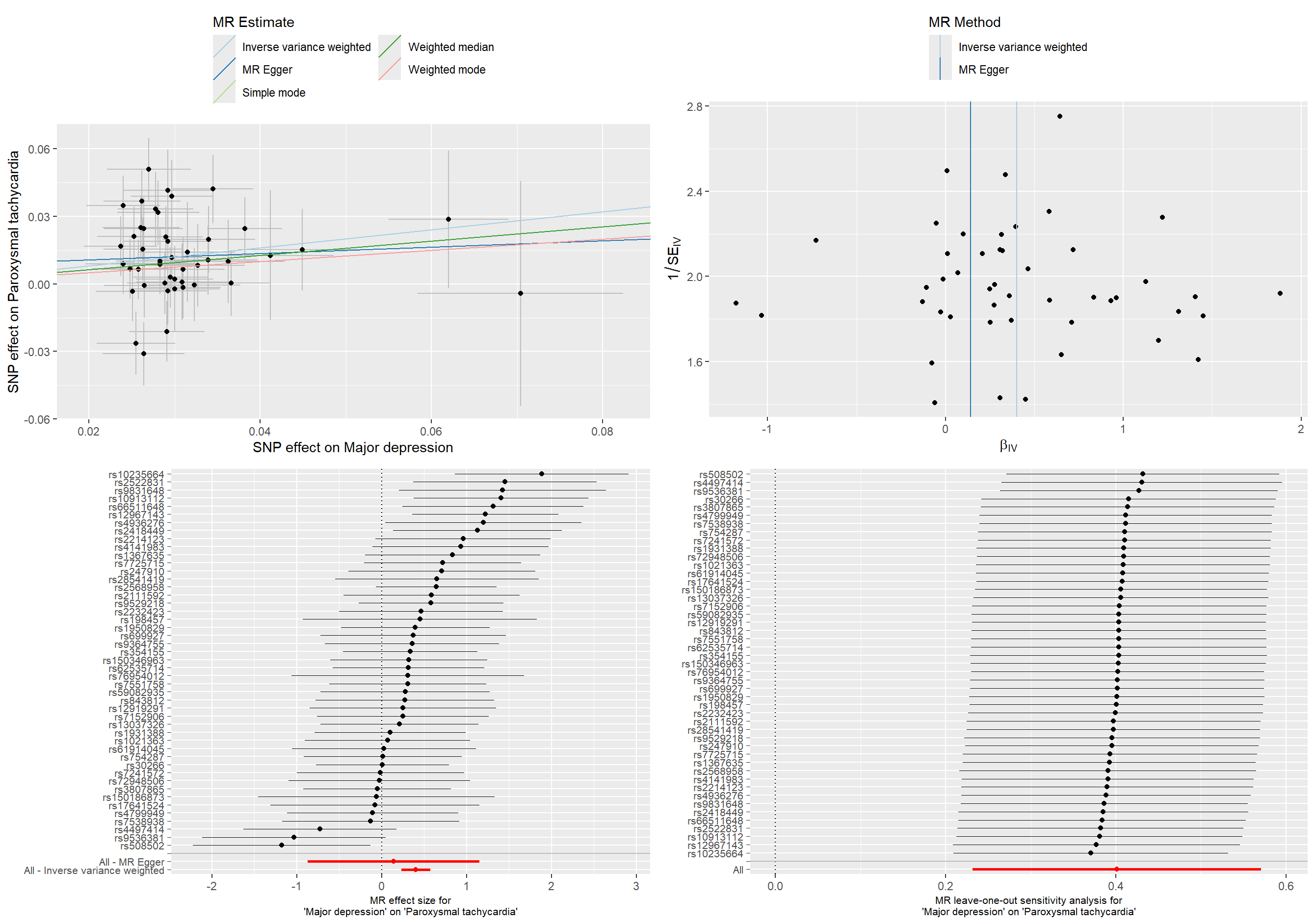
**

**Figure S3: The causal effect of** **Major depression on Paroxysmal tachycardia**

**
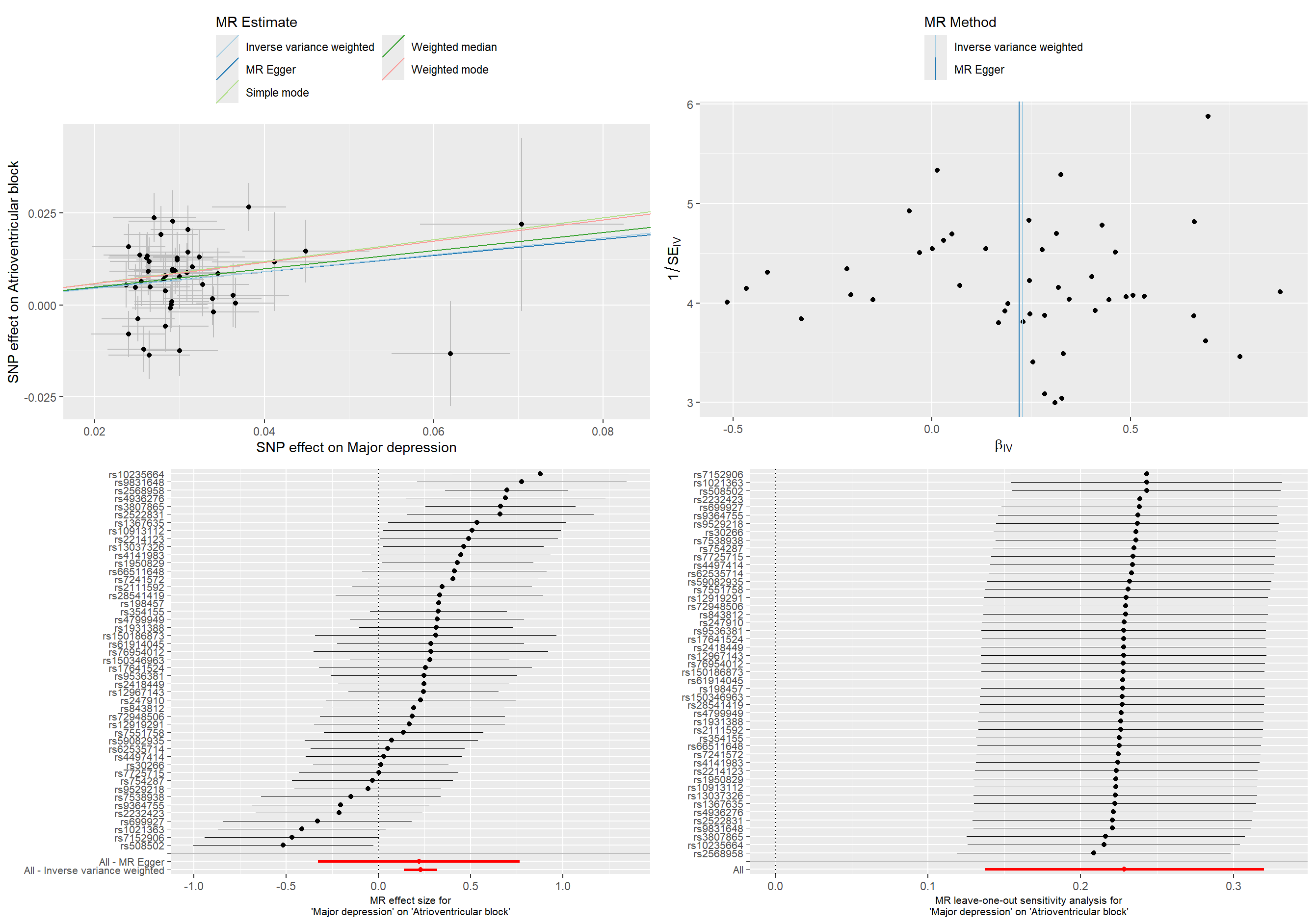
**

**Figure S4: The causal effect of** **Major depression on Atrioventricular block**

**
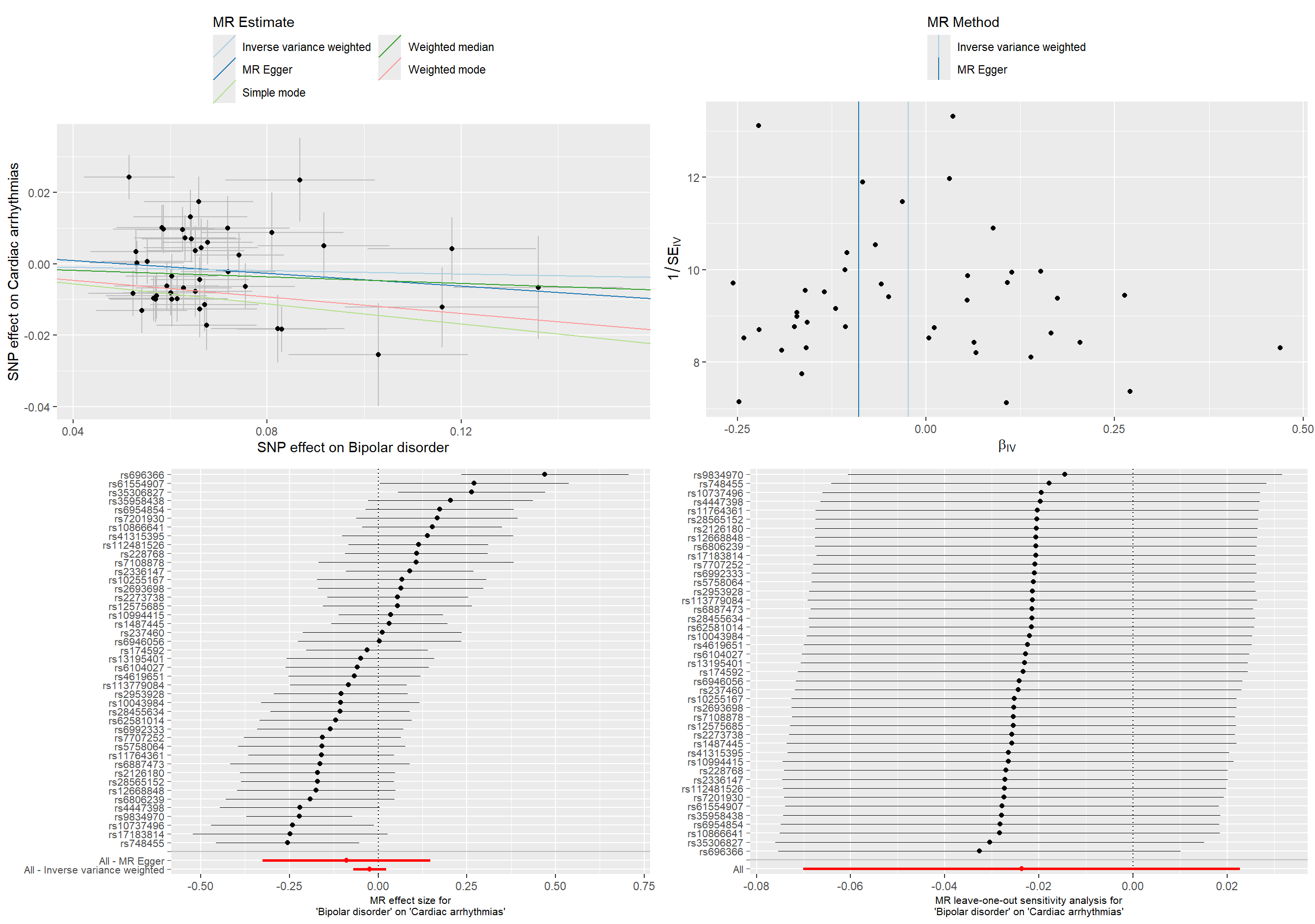
**

**Figure S5: The causal effect of** **Bipolar disorder on Cardiac arrhythmias**

**
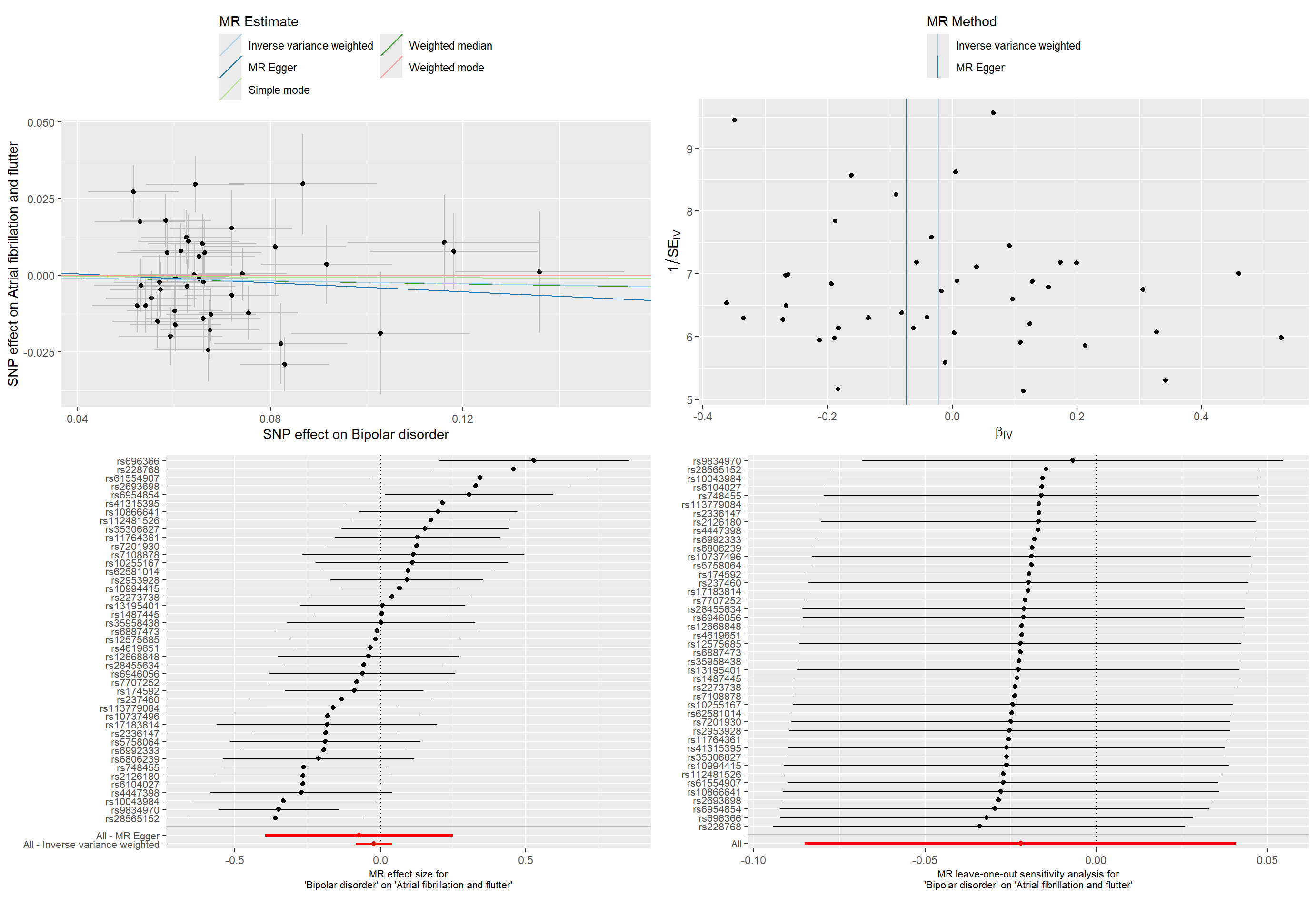
**

**Figure S6: The causal effect of** **Bipolar disorder on Atrial fibrillation and flutter**

**
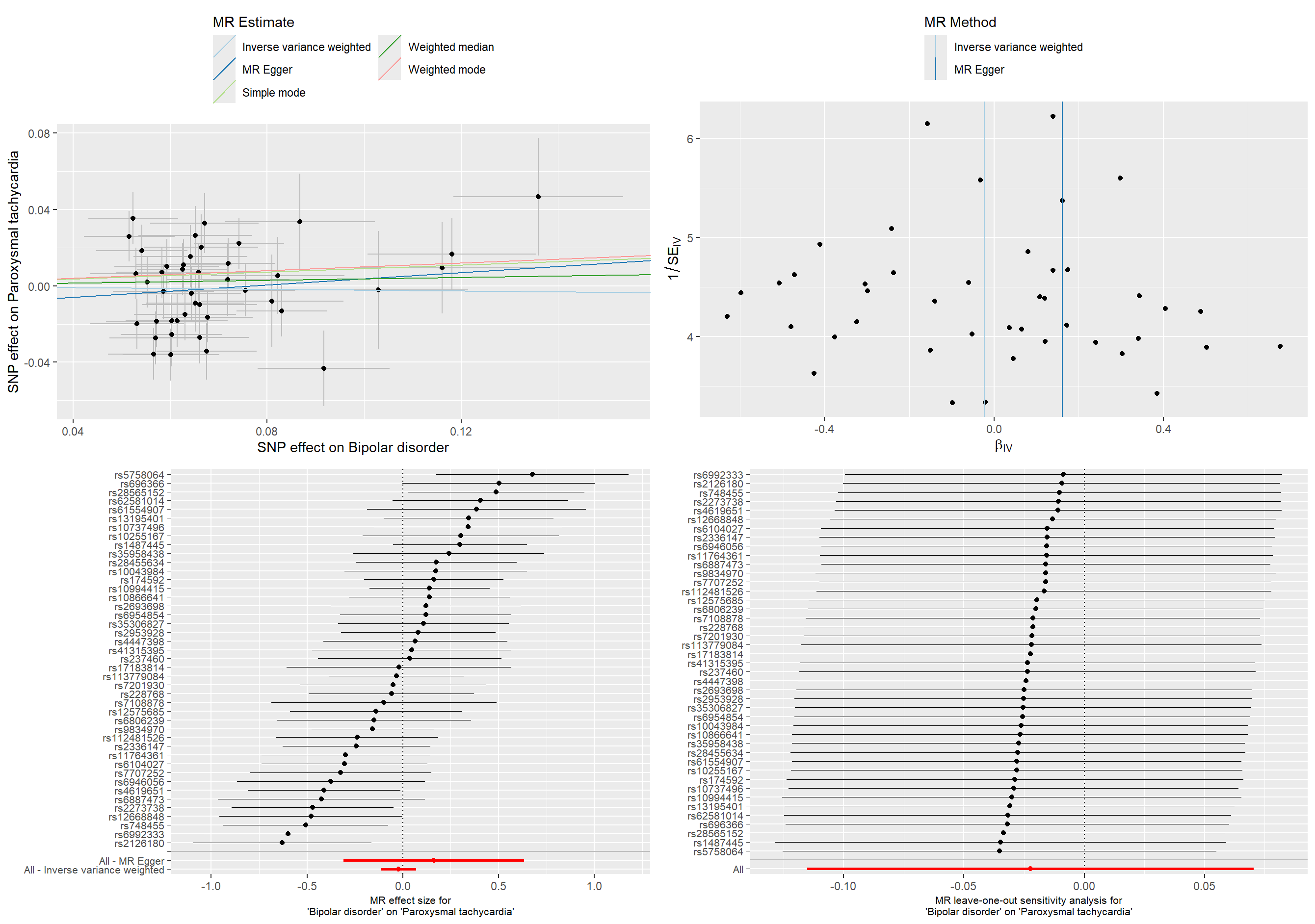
**

**Figure S7: The causal effect of** **Bipolar disorder on Paroxysmal tachycardia**

**
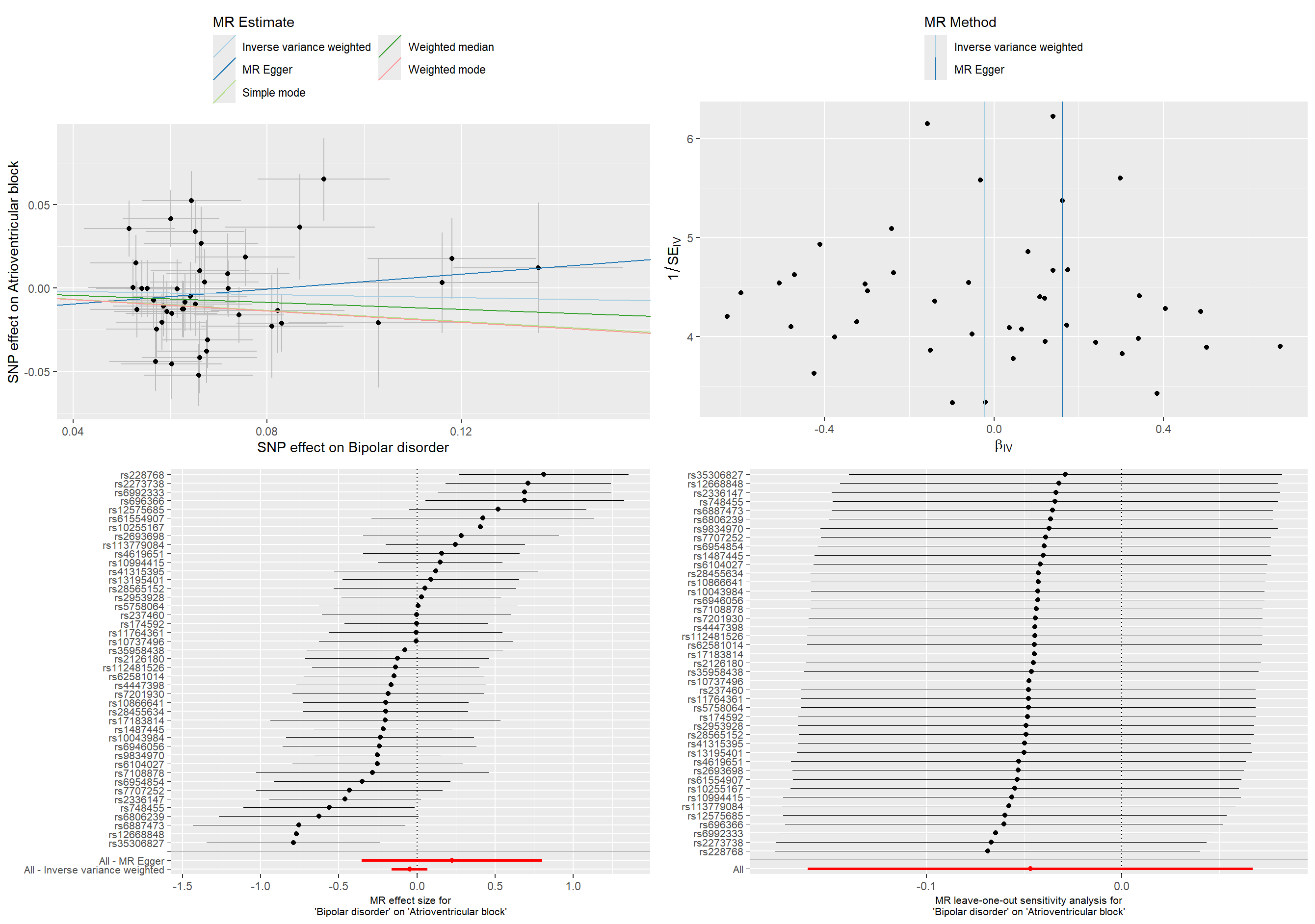
**

**Figure S8: The causal effect of** **Bipolar disorder on Atrioventricular block**

**
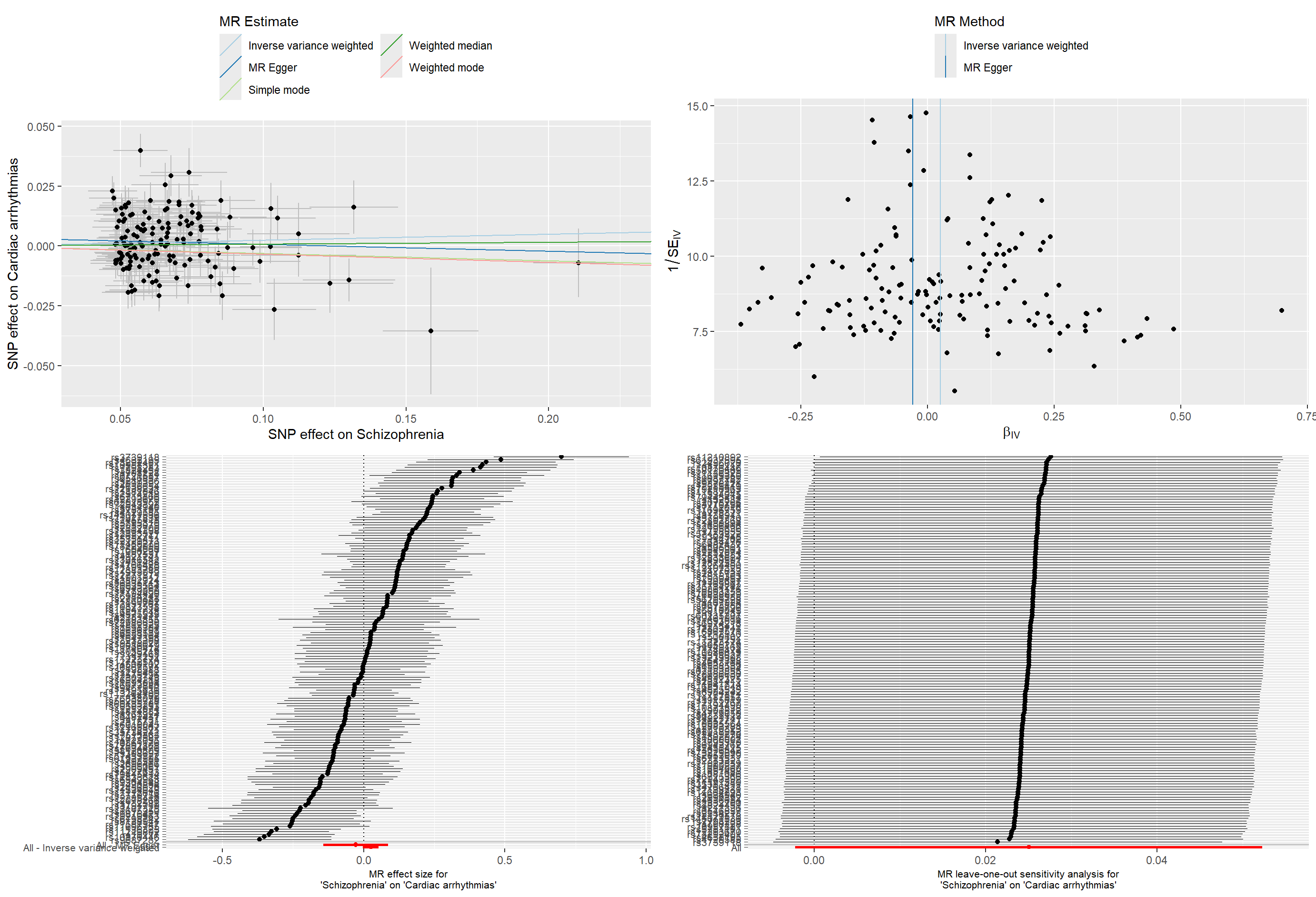
**

**Figure S9: The causal effect of** **Schizophrenia on Cardiac arrhythmias**

**
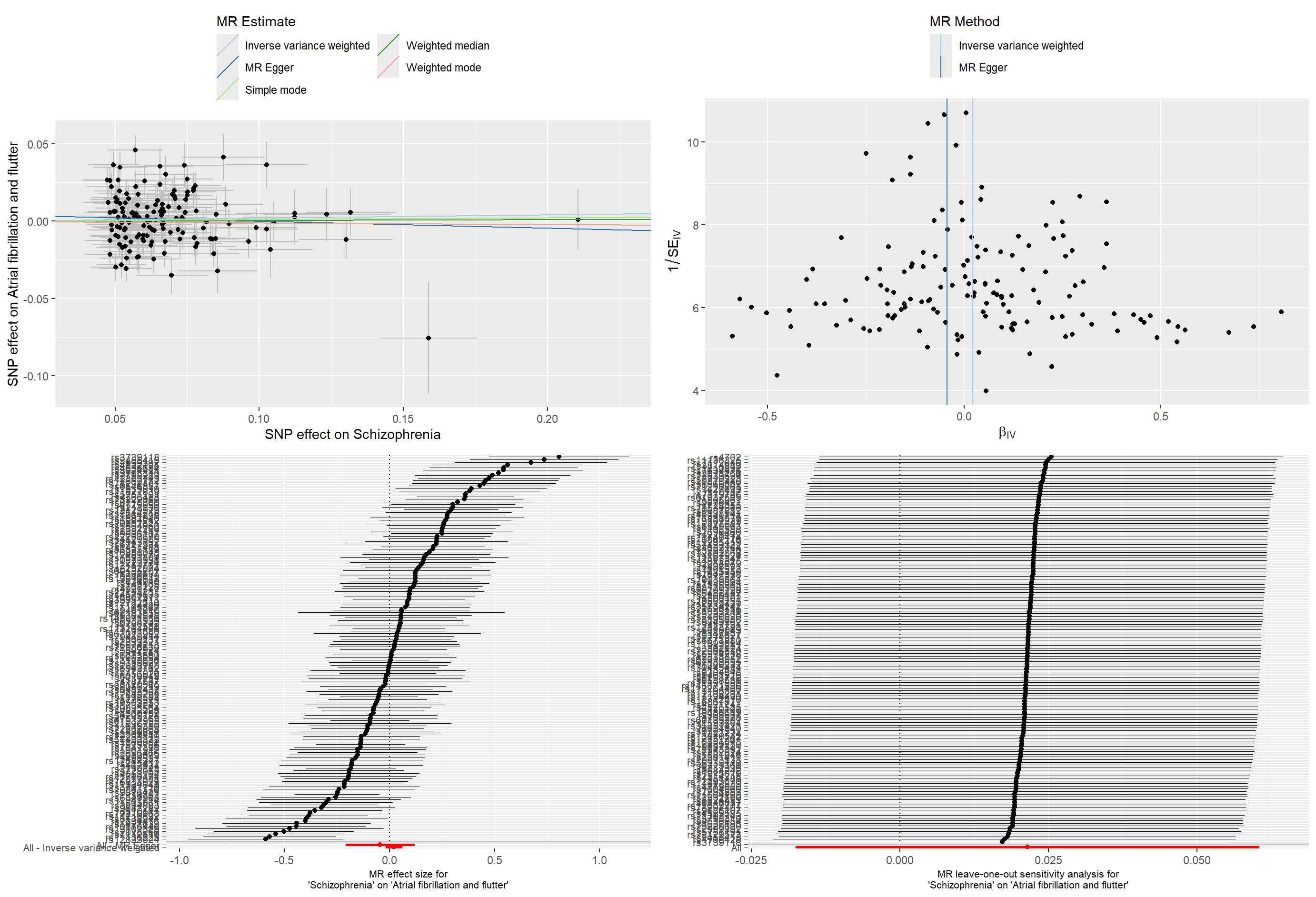
**

**Figure S10: The causal effect of** **Schizophrenia on Atrial fibrillation and flutter**

**
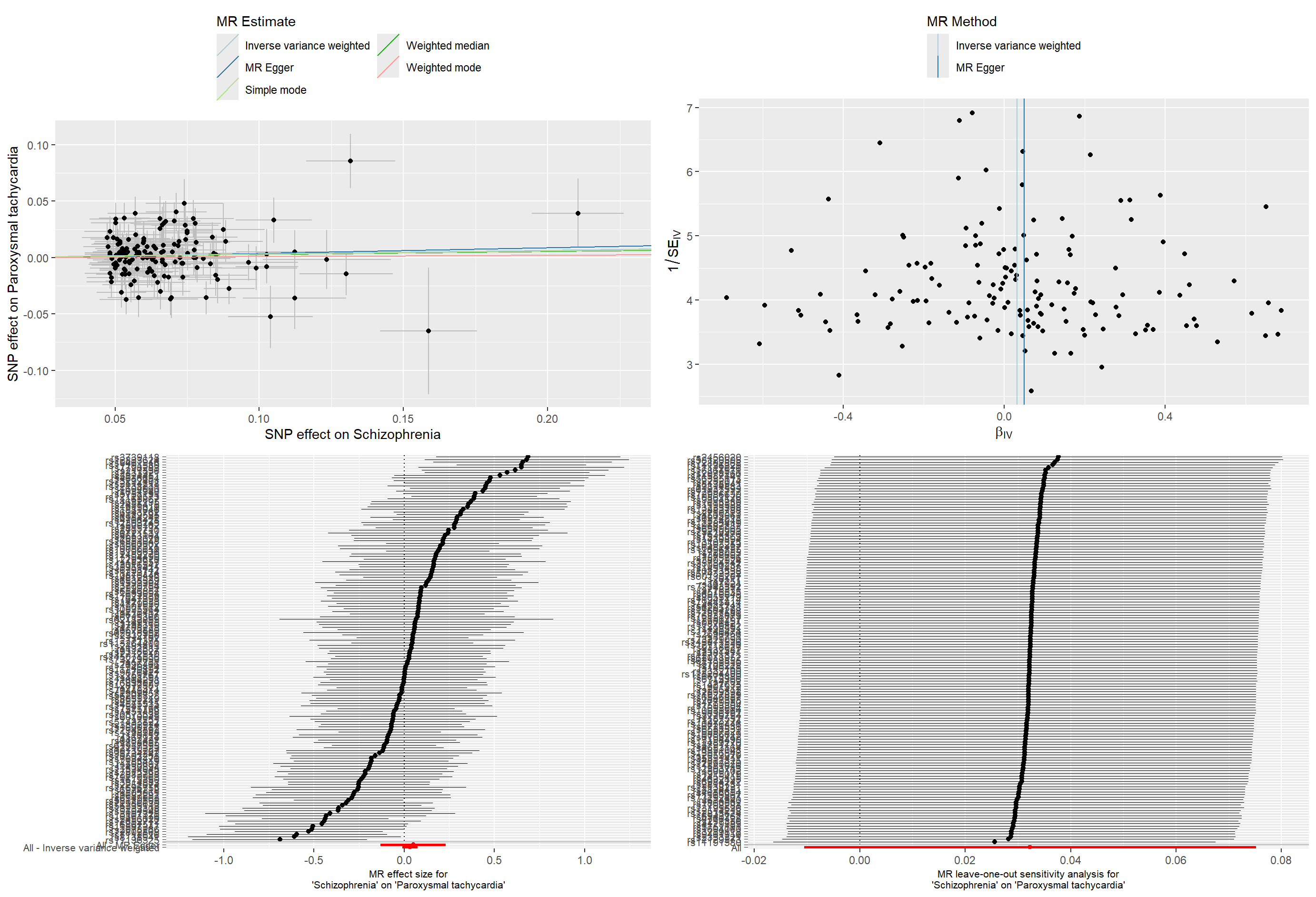
**

**Figure S11: The causal effect of** **Schizophrenia on Paroxysmal tachycardia**

**
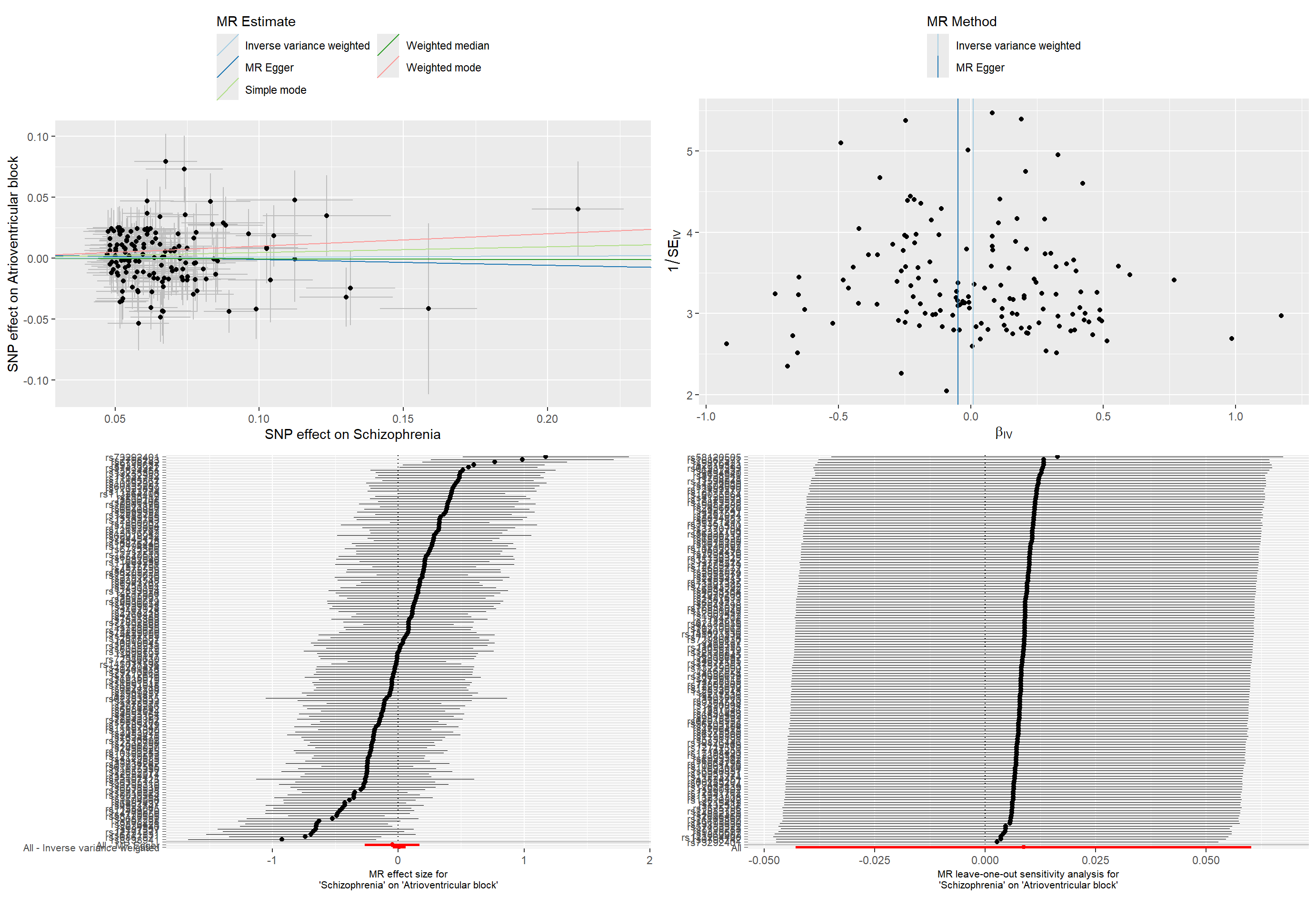
**

**Figure S12: The causal effect of** **Schizophrenia on Atrioventricular block**

**
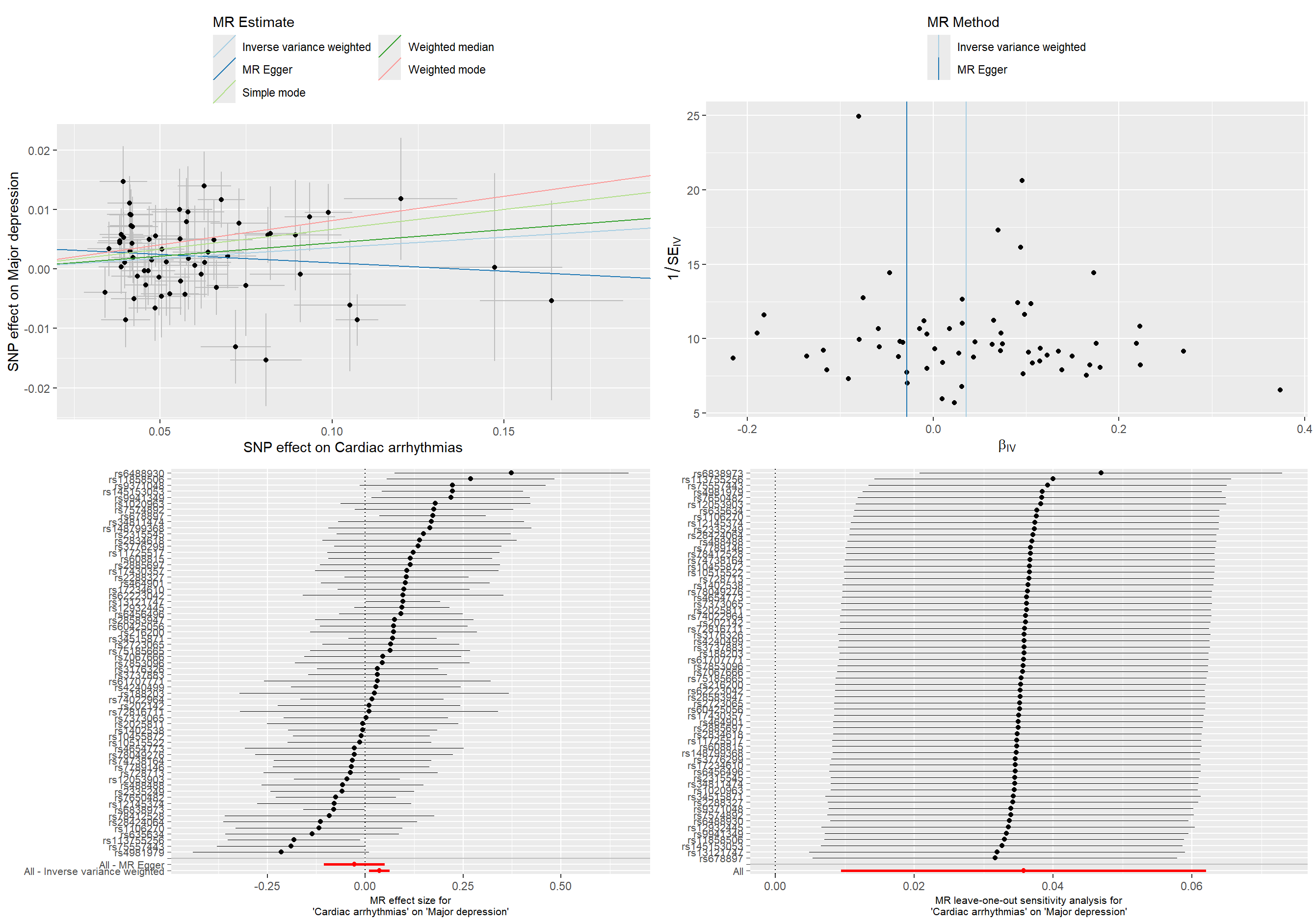
**

**Figure S13: The causal effect of** **Cardiac arrhythmias on Major depression**

**
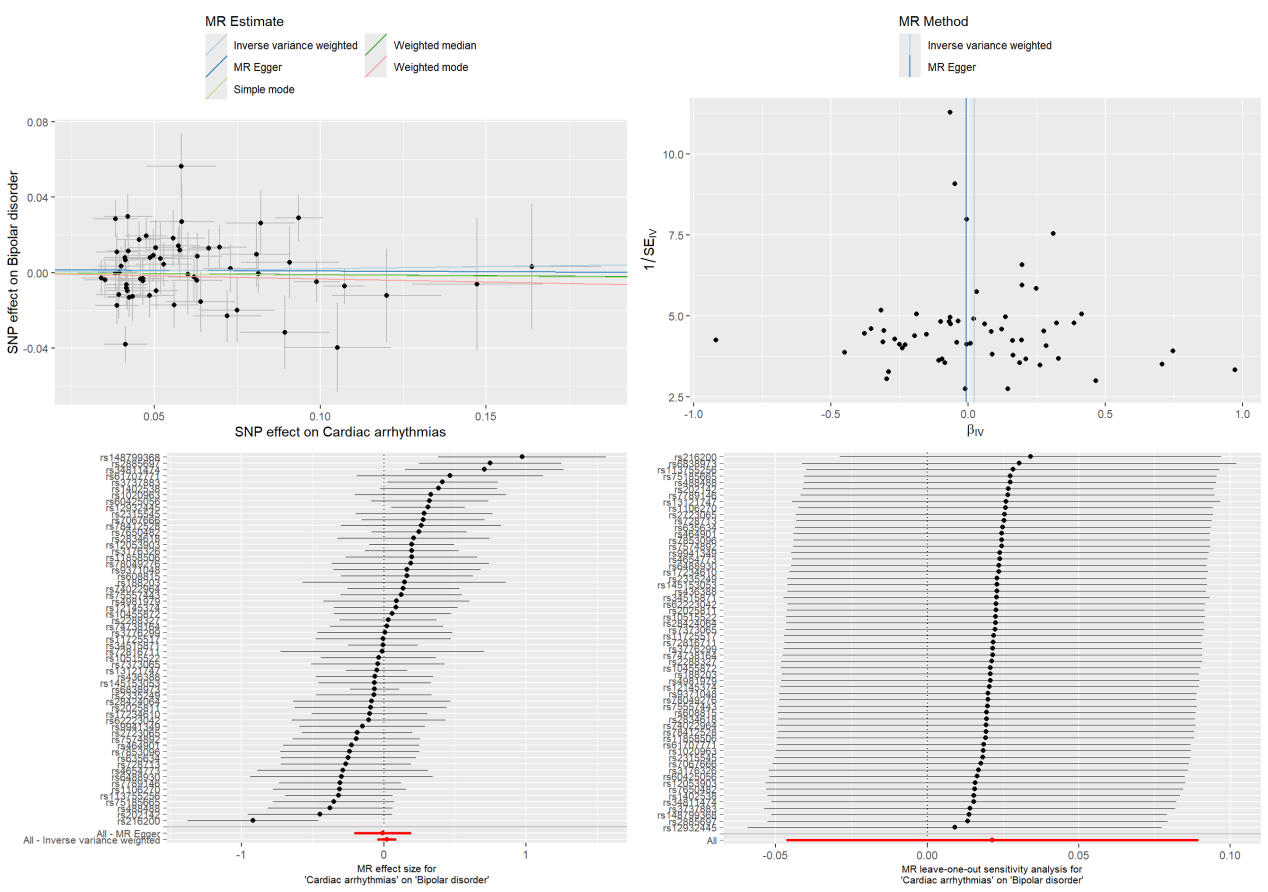
**

**Figure S14: The causal effect of** **Cardiac arrhythmias on Bipolar disorder**

**
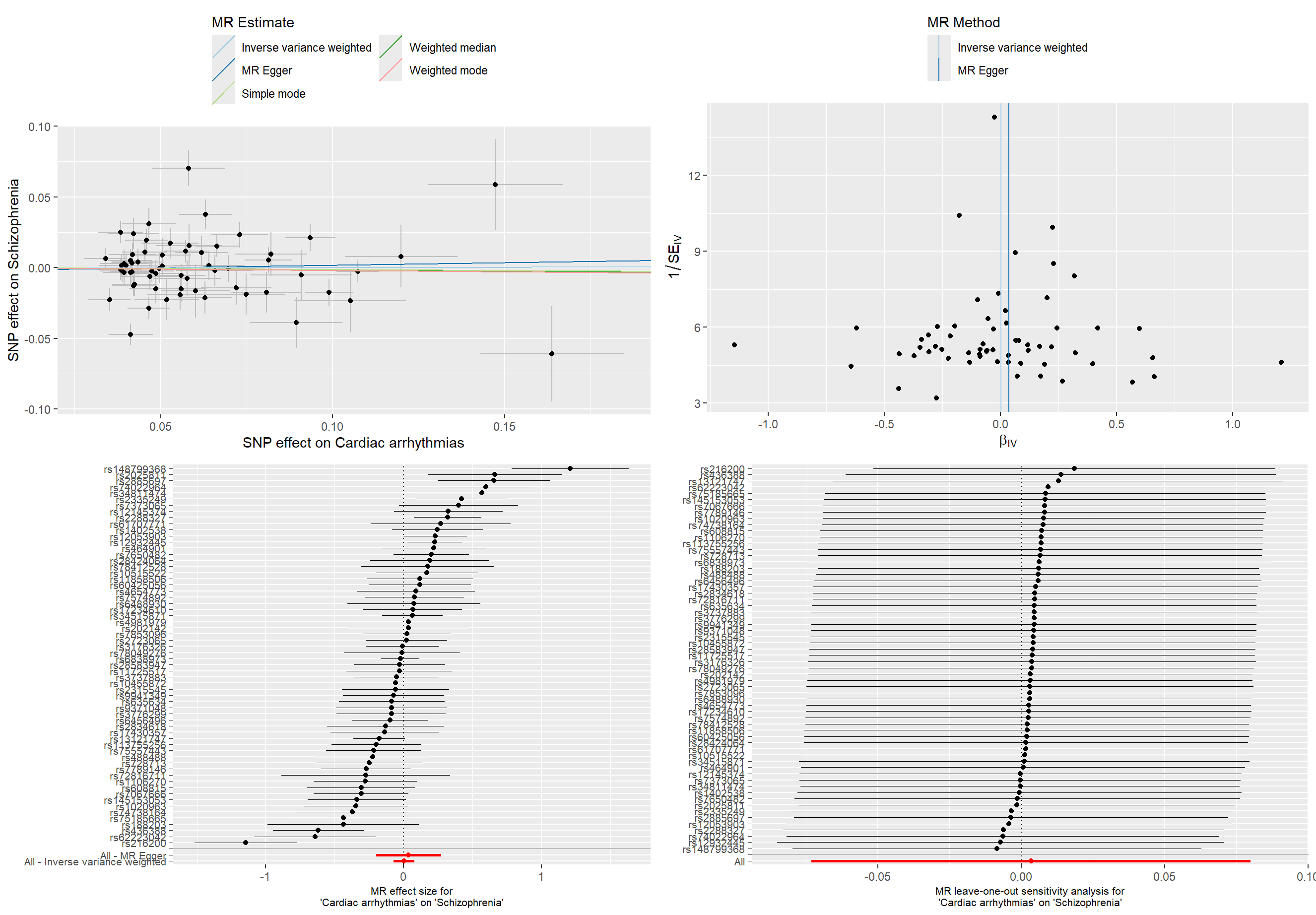
**

**Figure S15: The causal effect of** **Cardiac arrhythmias on Schizophrenia**

**
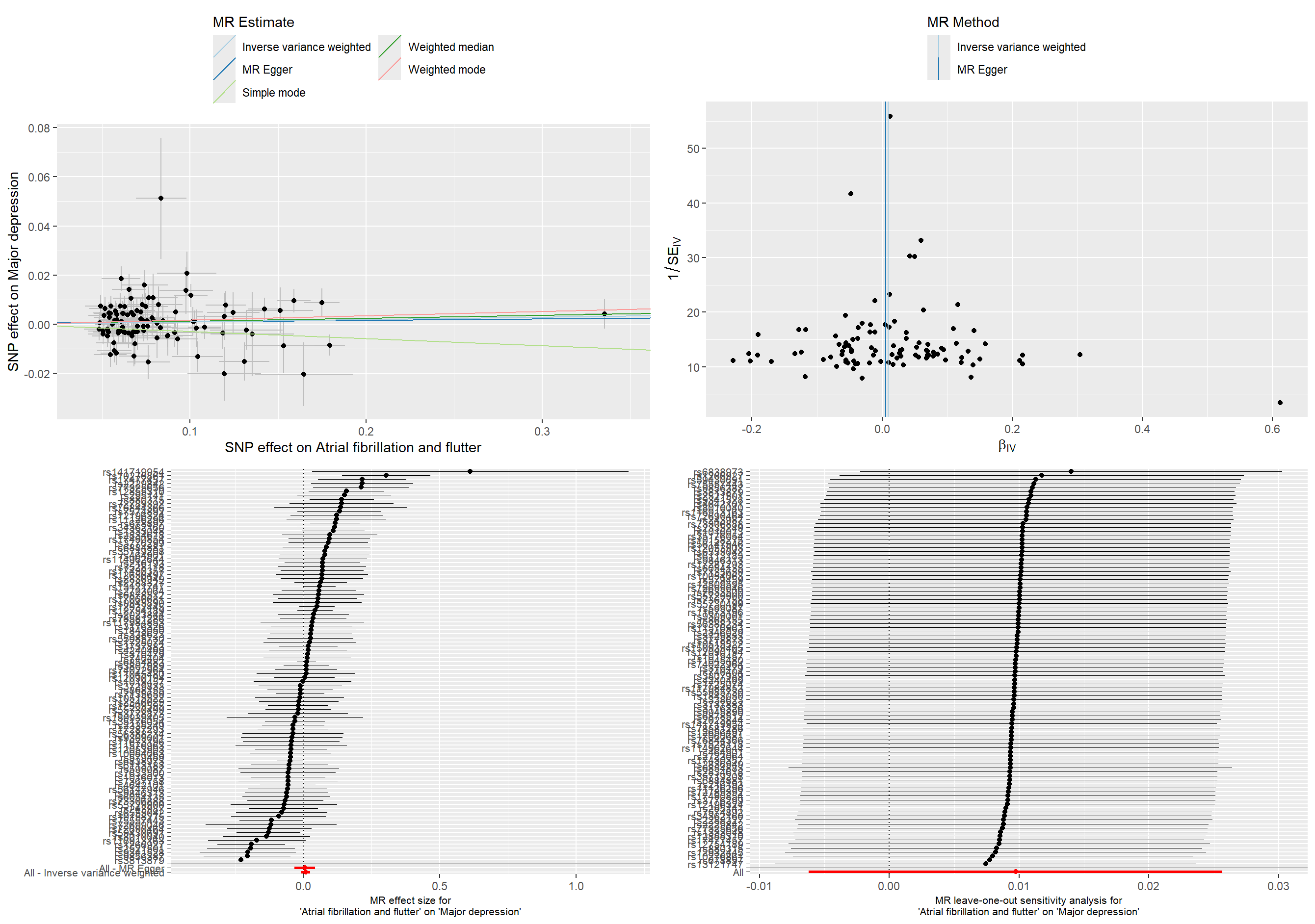
**

**Figure S16: The causal effect of** **Atrial fibrillation and flutter on Major depression**

**
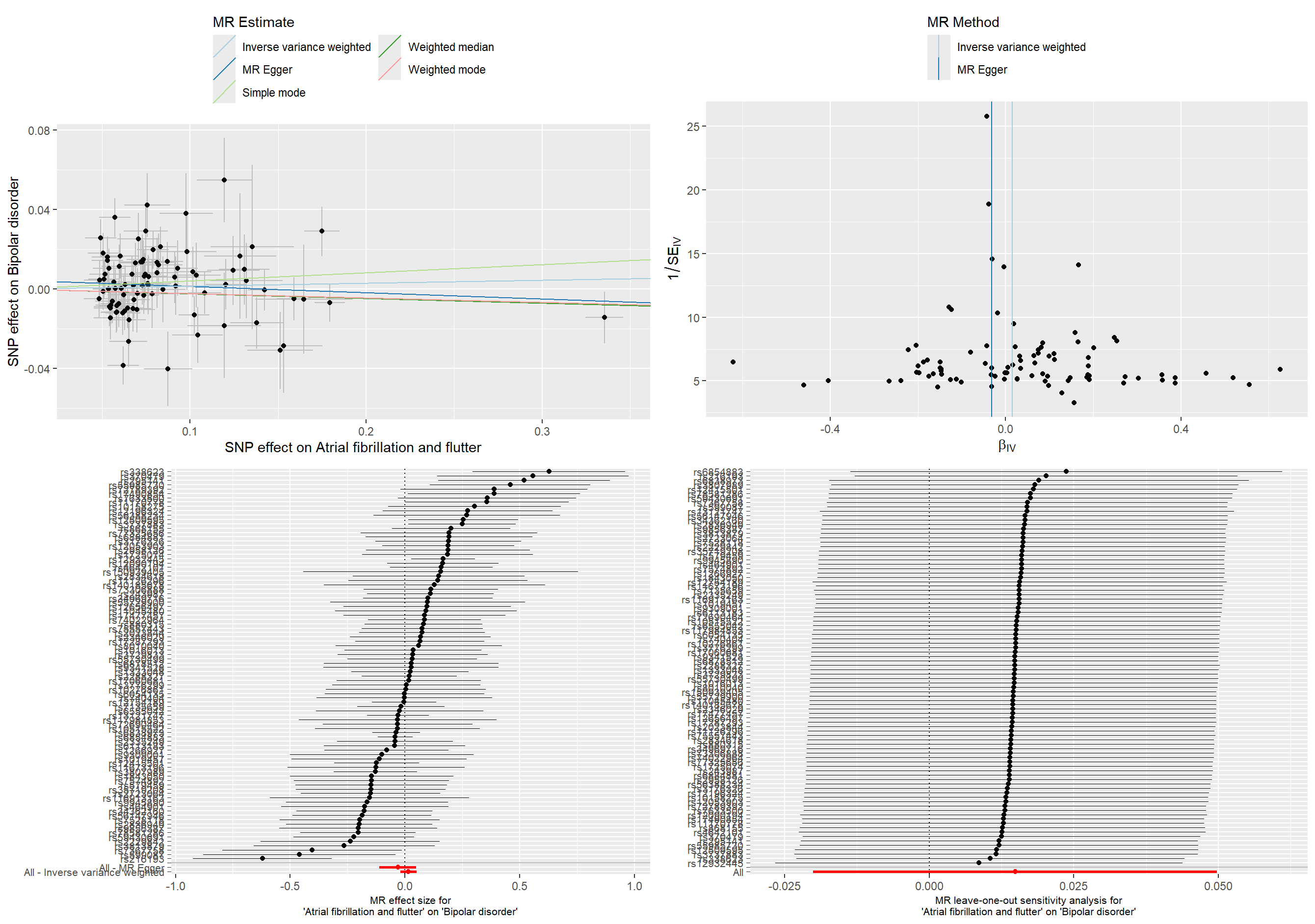
**

**Figure S17: The causal effect of** **Atrial fibrillation and flutter on Bipolar disorder**

**
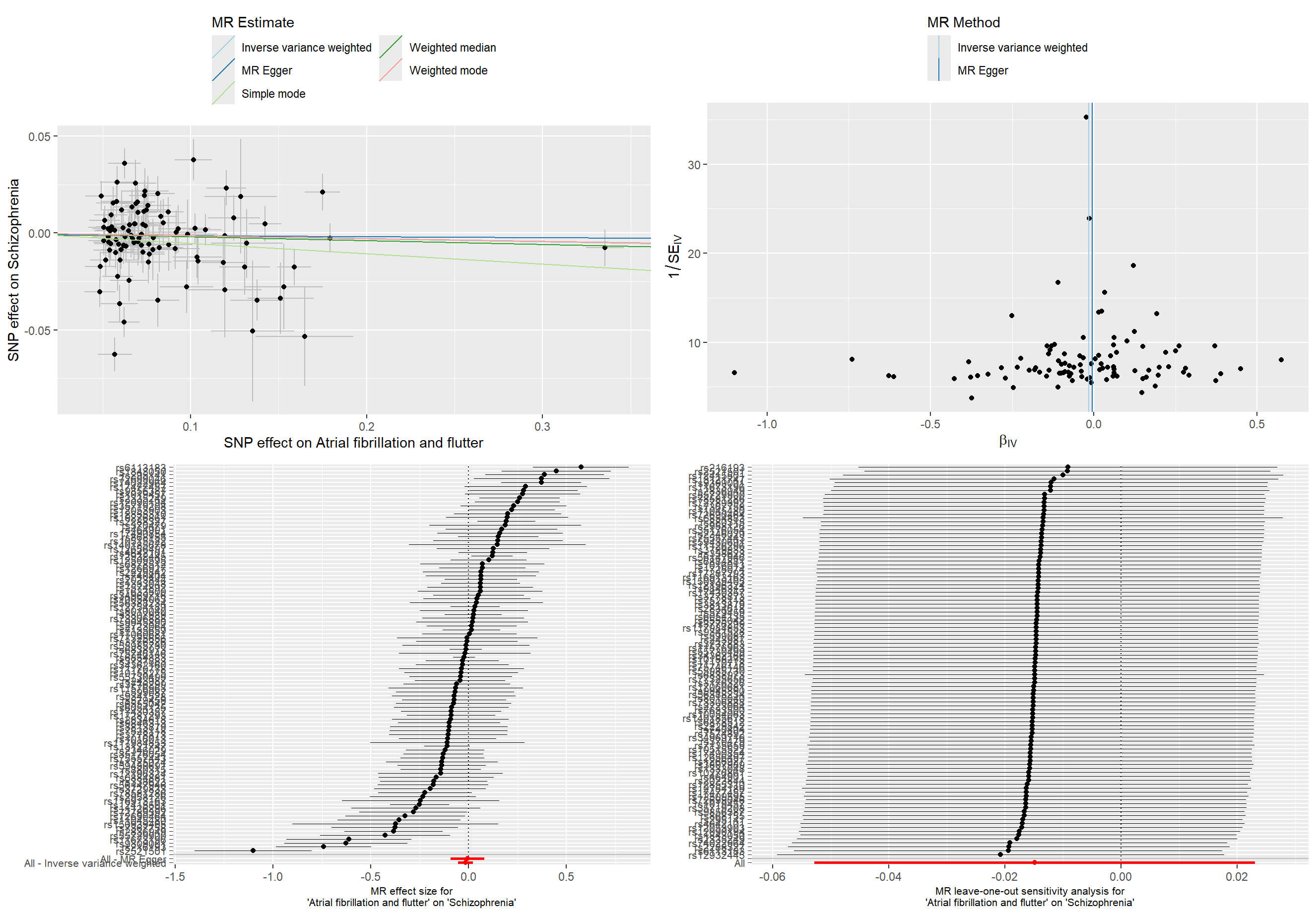
**

**Figure S18: The causal effect of** **Atrial fibrillation and flutter on Schizophrenia**

**
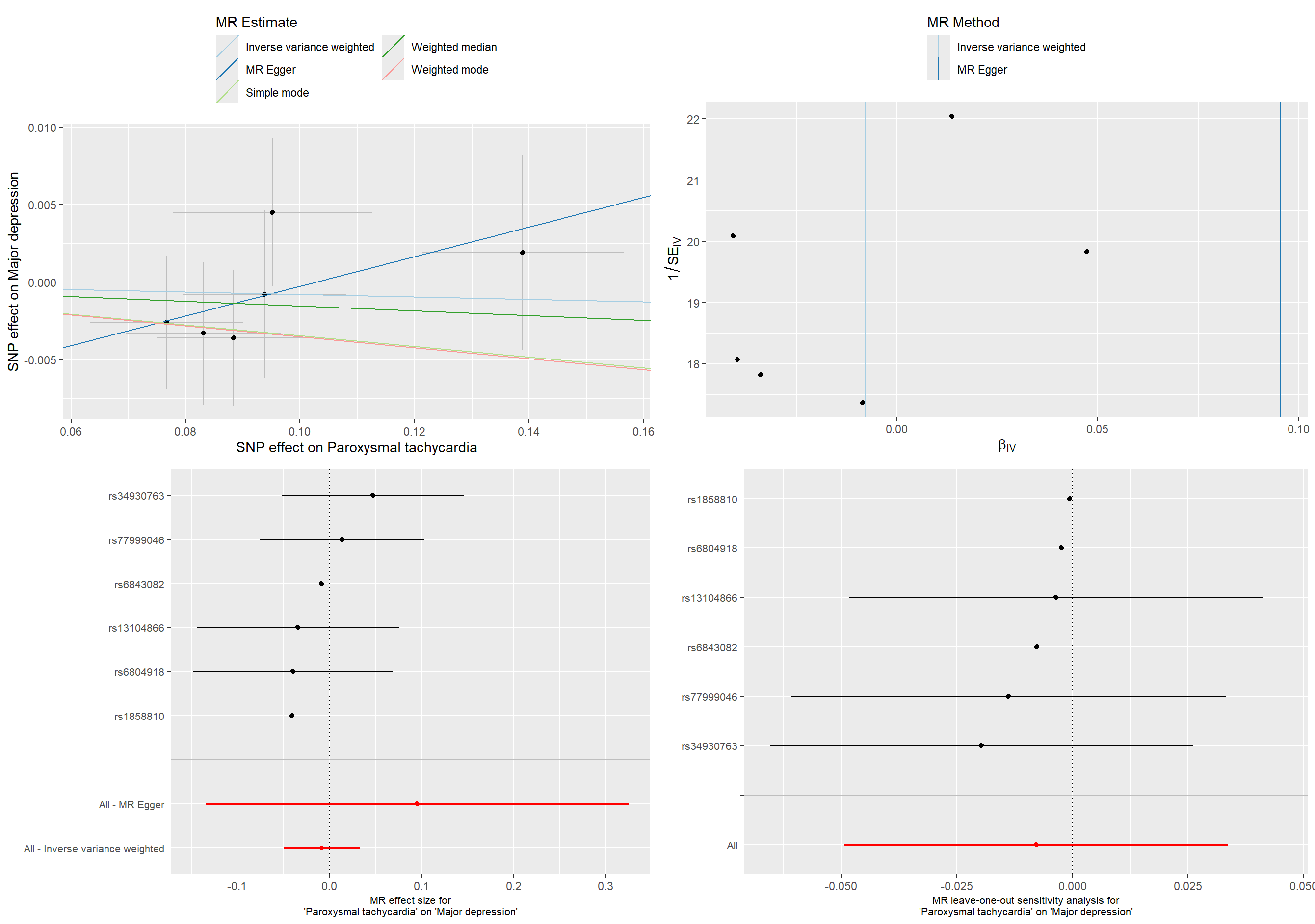
**

**Figure S19: The causal effect of** **Paroxysmal tachycardia on Major depression**

**
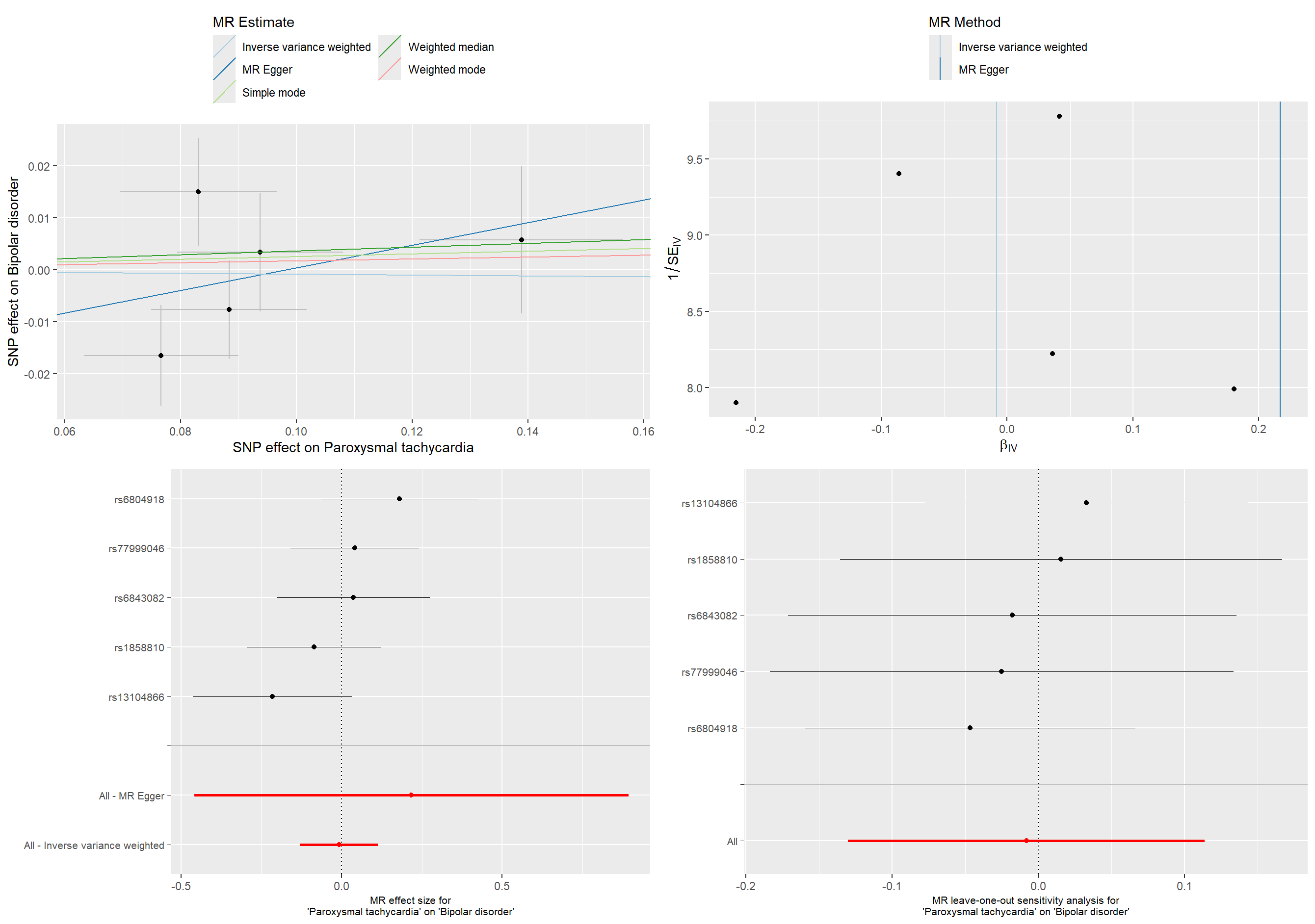
**

**Figure S20: The causal effect of** **Paroxysmal tachycardia on Bipolar disorder**

**
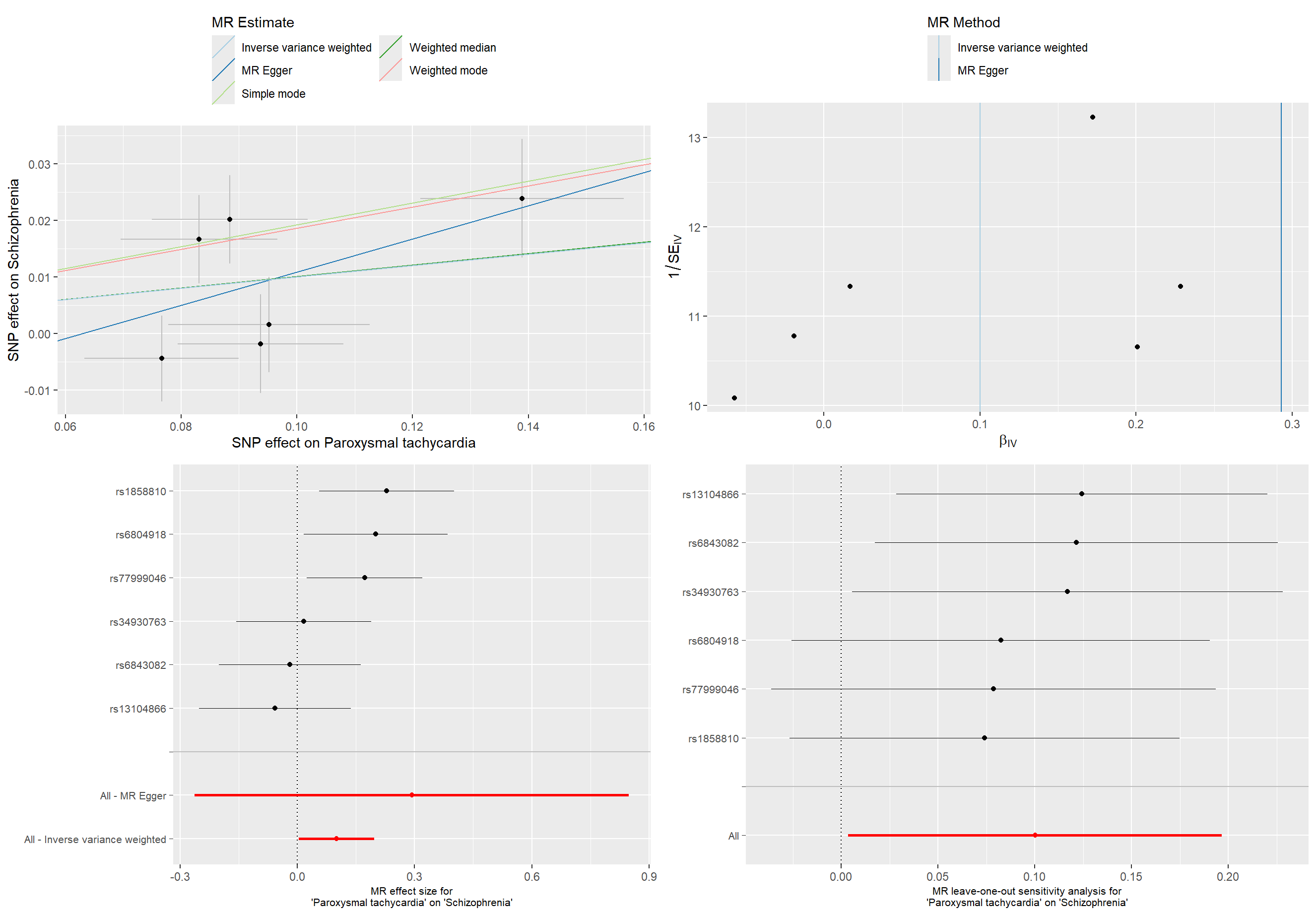
**

**Figure S21: The causal effect of** **Paroxysmal tachycardia on Schizophrenia**

**
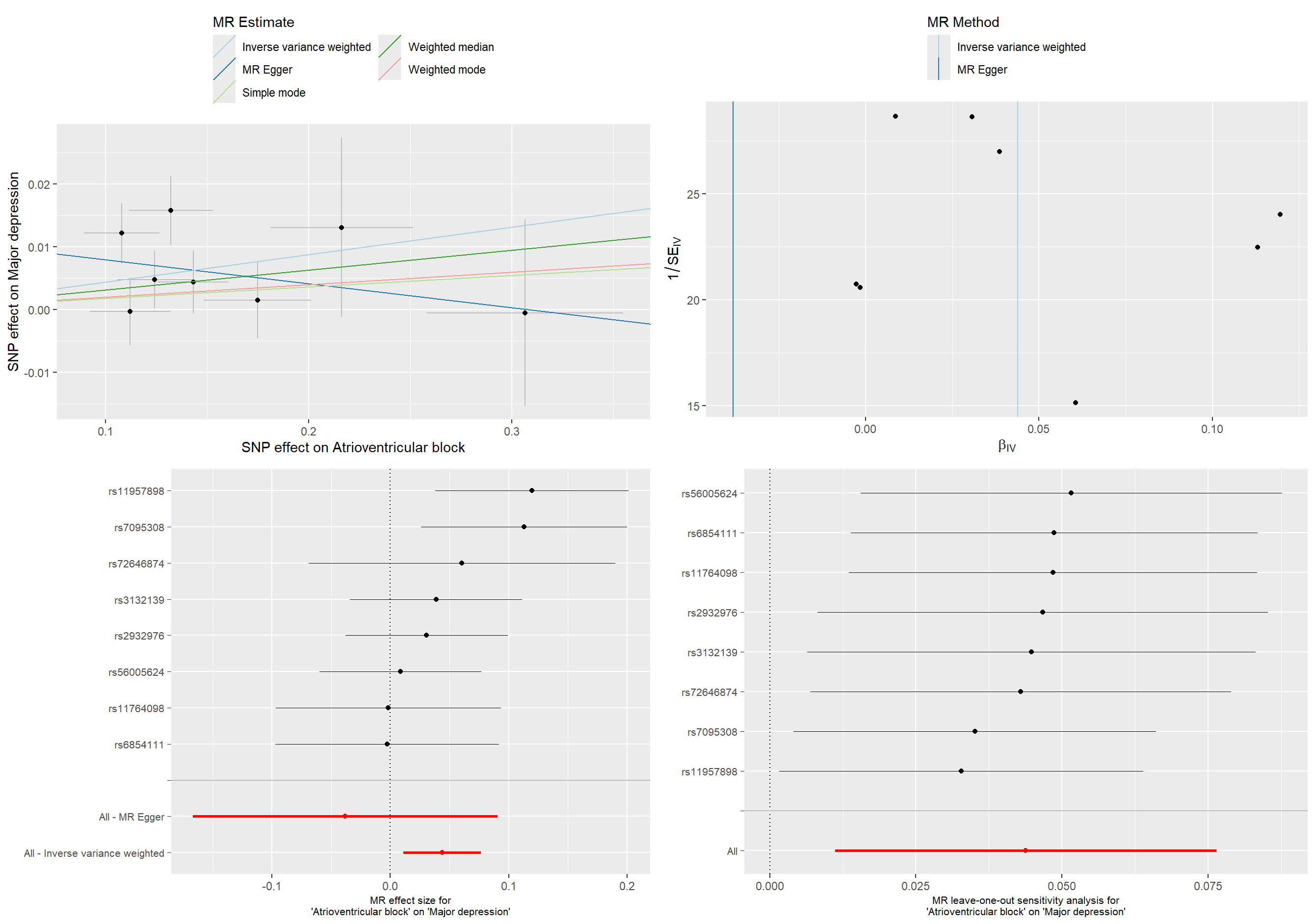
**

**Figure S22: The causal effect of** **Atrioventricular block on Major depression**

**
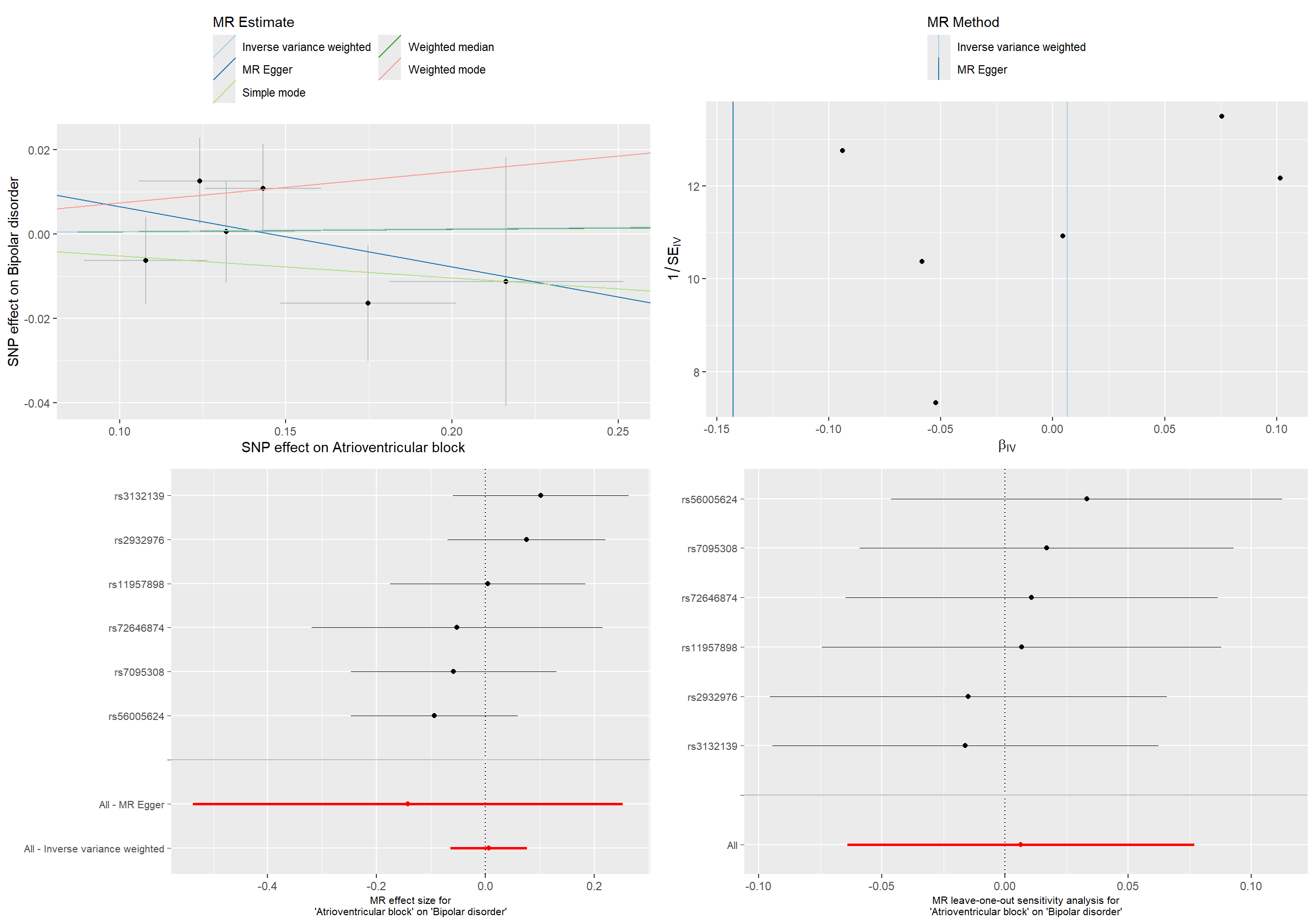
**

**Figure S23: The causal effect of** **Atrioventricular block on Bipolar disorder**

**
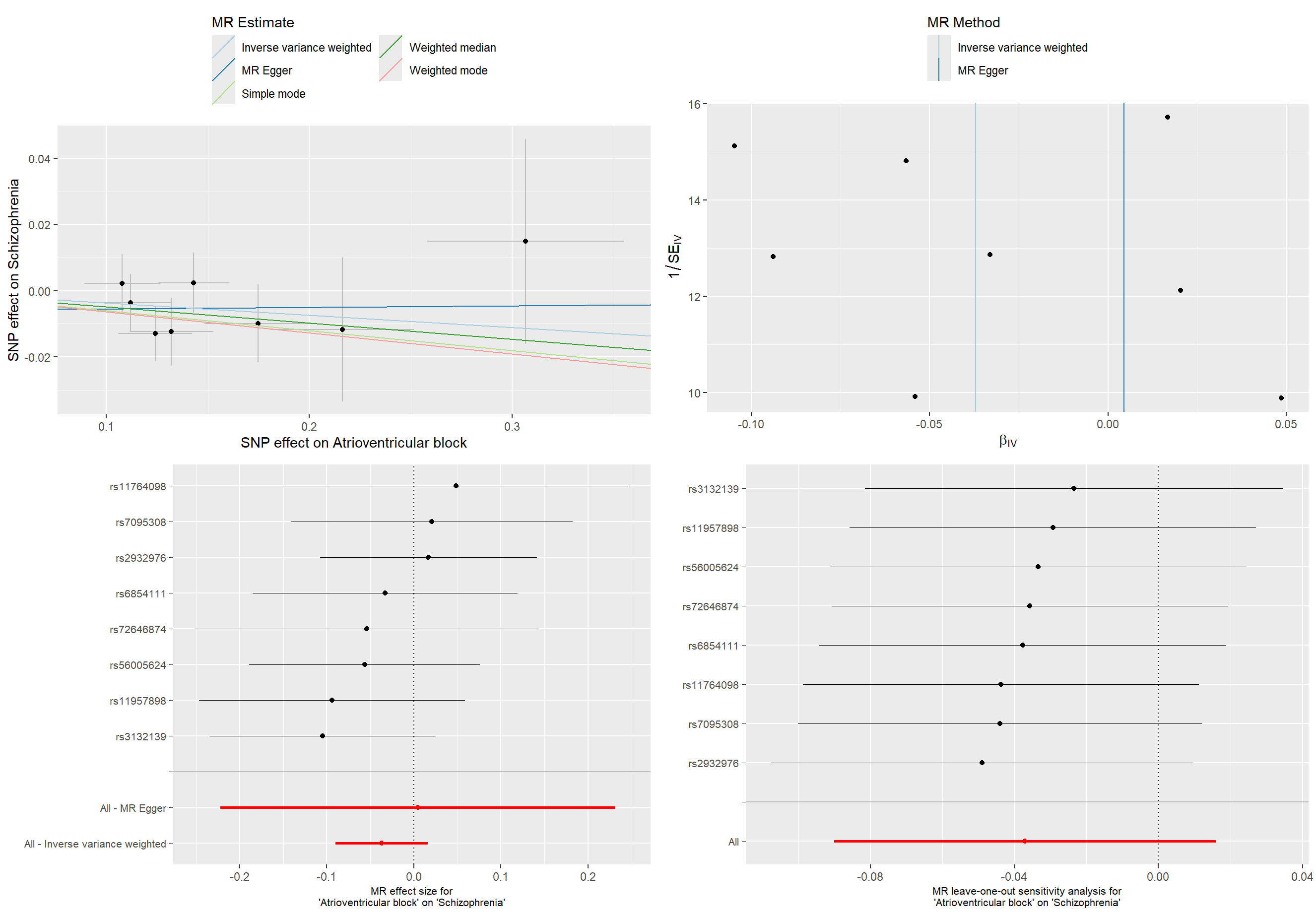
**

**Figure S24: The causal effect of** **Atrioventricular block on Schizophrenia**

**Table S1. 50 SNPs associated with depression.**

| SNP | Effect  allele | Other allele | Effect allele  frequency | Beta | Se | Pval | Sample  size | F |
| --- | --- | --- | --- | --- | --- | --- | --- | --- |
| rs4141983 | C | T | 0.326 | -0.026 | 0.005 | 9.69E-09 | 500,199 | 32.938 |
| rs354155 | C | G | 0.0923 | -0.045 | 0.008 | 1.75E-09 | 500,199 | 35.840 |
| rs7551758 | G | T | 0.5329 | 0.028 | 0.004 | 5.11E-11 | 500,199 | 43.315 |
| rs7538938 | C | T | 0.5599 | 0.025 | 0.004 | 7.29E-09 | 500,199 | 34.073 |
| rs2568958 | A | G | 0.6042 | 0.038 | 0.004 | 2.90E-18 | 500,199 | 75.374 |
| rs10913112 | T | C | 0.378 | -0.026 | 0.005 | 4.53E-09 | 500,199 | 33.898 |
| rs17641524 | T | C | 0.2101 | -0.030 | 0.005 | 1.50E-08 | 500,199 | 32.040 |
| rs2111592 | A | G | 0.3141 | 0.026 | 0.005 | 1.35E-08 | 500,199 | 32.689 |
| rs72948506 | A | G | 0.2975 | 0.027 | 0.005 | 1.71E-08 | 500,199 | 31.790 |
| rs9831648 | T | G | 0.7739 | -0.029 | 0.005 | 1.59E-08 | 500,199 | 31.533 |
| rs843812 | A | G | 0.4117 | 0.025 | 0.004 | 1.41E-08 | 500,199 | 31.769 |
| rs76954012 | A | T | 0.0931 | 0.041 | 0.007 | 2.41E-08 | 500,199 | 30.998 |
| rs66511648 | C | T | 0.284 | 0.030 | 0.005 | 6.03E-10 | 500,199 | 38.285 |
| rs699927 | G | T | 0.4171 | 0.024 | 0.004 | 3.79E-08 | 500,199 | 29.752 |
| rs247910 | G | A | 0.457 | 0.024 | 0.004 | 4.71E-08 | 500,199 | 30.378 |
| rs30266 | A | G | 0.3271 | 0.037 | 0.005 | 1.43E-15 | 500,199 | 63.306 |
| rs7725715 | A | G | 0.5343 | 0.029 | 0.004 | 1.61E-11 | 500,199 | 45.484 |
| rs150186873 | C | A | 0.0327 | 0.070 | 0.012 | 4.51E-09 | 500,199 | 34.418 |
| rs2232423 | G | A | 0.1056 | -0.062 | 0.007 | 1.14E-18 | 500,199 | 78.449 |
| rs2214123 | G | A | 0.6466 | -0.026 | 0.005 | 8.56E-09 | 500,199 | 33.640 |
| rs2876520 | G | C | 0.4688 | 0.026 | 0.004 | 2.24E-09 | 500,199 | 36.560 |
| rs9364755 | G | A | 0.2262 | 0.028 | 0.005 | 3.49E-08 | 500,199 | 30.792 |
| rs10235664 | C | T | 0.2529 | -0.027 | 0.005 | 4.68E-08 | 500,199 | 30.362 |
| rs3807865 | A | G | 0.4105 | 0.031 | 0.004 | 1.09E-12 | 500,199 | 49.638 |
| rs59082935 | T | C | 0.1342 | 0.036 | 0.007 | 3.07E-08 | 500,199 | 30.250 |
| rs2522831 | C | T | 0.4739 | 0.024 | 0.004 | 2.11E-08 | 500,199 | 31.152 |
| rs4730387 | A | T | 0.4659 | 0.024 | 0.004 | 4.12E-08 | 500,199 | 30.635 |
| rs150346963 | T | C | 0.4118 | 0.028 | 0.004 | 1.16E-10 | 500,199 | 41.368 |
| rs1931388 | G | A | 0.4042 | -0.030 | 0.004 | 1.68E-11 | 500,199 | 44.951 |
| rs59283172 | A | G | 0.1081 | -0.039 | 0.007 | 2.41E-08 | 500,199 | 31.041 |
| rs62535714 | A | G | 0.1639 | 0.034 | 0.006 | 4.69E-09 | 500,199 | 34.162 |
| rs2418449 | C | T | 0.281 | -0.028 | 0.005 | 4.25E-09 | 500,199 | 34.271 |
| rs1021363 | G | A | 0.6434 | -0.030 | 0.005 | 2.29E-11 | 500,199 | 44.444 |
| rs198457 | T | C | 0.1886 | -0.032 | 0.006 | 1.90E-08 | 500,199 | 31.641 |
| rs4497414 | C | T | 0.44 | 0.029 | 0.004 | 2.93E-11 | 500,199 | 43.740 |
| rs4936276 | C | G | 0.622 | 0.028 | 0.004 | 3.57E-10 | 500,199 | 39.919 |
| rs61914045 | A | G | 0.2034 | 0.031 | 0.005 | 7.96E-09 | 500,199 | 32.744 |
| rs9529218 | T | C | 0.2031 | -0.034 | 0.005 | 2.23E-10 | 500,199 | 39.643 |
| rs9536381 | T | C | 0.3259 | 0.026 | 0.005 | 2.62E-08 | 500,199 | 30.730 |
| rs508502 | T | C | 0.2992 | -0.026 | 0.005 | 3.56E-08 | 500,199 | 30.250 |
| rs1950829 | G | A | 0.5173 | -0.030 | 0.004 | 4.74E-12 | 500,199 | 47.706 |
| rs7152906 | C | T | 0.5196 | 0.026 | 0.004 | 1.87E-09 | 500,199 | 36.000 |
| rs754287 | A | T | 0.3664 | -0.029 | 0.005 | 1.31E-10 | 500,199 | 41.245 |
| rs28541419 | G | C | 0.2308 | -0.029 | 0.005 | 1.76E-08 | 500,199 | 31.533 |
| rs12919291 | C | G | 0.1884 | 0.033 | 0.006 | 3.09E-09 | 500,199 | 35.348 |
| rs4799949 | T | C | 0.6684 | -0.029 | 0.005 | 1.40E-10 | 500,199 | 40.295 |
| rs1367635 | C | T | 0.5148 | 0.025 | 0.004 | 4.35E-09 | 500,199 | 34.618 |
| rs12967143 | C | G | 0.7012 | -0.035 | 0.005 | 2.53E-13 | 500,199 | 53.882 |
| rs7241572 | A | G | 0.2047 | 0.032 | 0.005 | 2.43E-09 | 500,199 | 35.778 |
| rs13037326 | T | C | 0.2597 | 0.031 | 0.005 | 2.40E-10 | 500,199 | 40.025 |

**Table S2. 52 SNPs associated with bipolar disorder.**

| SNP | Effect  allele | Other allele | Beta | Se | Pval | Sample  size | F |
| --- | --- | --- | --- | --- | --- | --- | --- |
| rs2126180 | A | G | 0.057 | 0.009 | 1.62E-09 | 413,466 | 36.258 |
| rs10737496 | T | C | -0.054 | 0.009 | 7.17E-09 | 413,466 | 33.251 |
| rs4619651 | A | G | -0.066 | 0.010 | 4.78E-11 | 413,466 | 42.827 |
| rs17183814 | A | G | -0.103 | 0.019 | 2.68E-08 | 413,466 | 30.937 |
| rs13417268 | G | C | -0.062 | 0.011 | 2.05E-08 | 413,466 | 31.564 |
| rs2011302 | A | T | 0.053 | 0.010 | 4.25E-08 | 413,466 | 29.748 |
| rs9834970 | C | T | 0.083 | 0.009 | 6.63E-19 | 413,466 | 79.653 |
| rs2336147 | C | T | -0.068 | 0.009 | 3.61E-13 | 413,466 | 52.986 |
| rs6806239 | G | T | -0.066 | 0.012 | 2.64E-08 | 413,466 | 30.851 |
| rs696366 | A | C | -0.052 | 0.009 | 4.46E-08 | 413,466 | 30.128 |
| rs112481526 | G | A | 0.063 | 0.011 | 1.86E-09 | 413,466 | 36.114 |
| rs28565152 | A | G | 0.067 | 0.011 | 1.96E-09 | 413,466 | 35.895 |
| rs7707252 | G | A | 0.057 | 0.010 | 3.64E-08 | 413,466 | 30.245 |
| rs6887473 | A | G | -0.060 | 0.011 | 8.81E-09 | 413,466 | 32.985 |
| rs10043984 | T | C | 0.059 | 0.011 | 3.71E-08 | 413,466 | 30.153 |
| rs10866641 | C | T | -0.063 | 0.009 | 2.79E-11 | 413,466 | 44.349 |
| rs13195401 | T | G | -0.136 | 0.018 | 6.98E-15 | 413,466 | 60.308 |
| rs41315395 | A | C | 0.072 | 0.013 | 1.48E-08 | 413,466 | 32.055 |
| rs1487445 | T | C | 0.074 | 0.009 | 1.48E-15 | 413,466 | 63.649 |
| rs10455979 | G | C | 0.056 | 0.010 | 4.22E-09 | 413,466 | 34.250 |
| rs12668848 | A | G | -0.057 | 0.010 | 1.90E-09 | 413,466 | 36.005 |
| rs113779084 | A | G | 0.075 | 0.010 | 1.42E-13 | 413,466 | 54.789 |
| rs6954854 | A | G | -0.058 | 0.009 | 5.94E-10 | 413,466 | 38.463 |
| rs11764361 | G | A | -0.061 | 0.010 | 3.47E-09 | 413,466 | 34.968 |
| rs6946056 | C | A | 0.053 | 0.010 | 3.66E-08 | 413,466 | 30.081 |
| rs10255167 | A | G | 0.066 | 0.012 | 1.60E-08 | 413,466 | 31.661 |
| rs62489493 | G | C | 0.090 | 0.014 | 2.64E-11 | 413,466 | 44.144 |
| rs2953928 | A | G | 0.116 | 0.020 | 6.25E-09 | 413,466 | 33.696 |
| rs6992333 | G | A | 0.060 | 0.010 | 1.62E-09 | 413,466 | 36.236 |
| rs62581014 | T | C | 0.065 | 0.012 | 2.77E-08 | 413,466 | 31.055 |
| rs1998820 | A | T | -0.084 | 0.015 | 4.10E-08 | 413,466 | 30.321 |
| rs10994415 | C | T | 0.118 | 0.017 | 1.14E-11 | 413,466 | 46.066 |
| rs2273738 | T | C | 0.092 | 0.014 | 1.63E-11 | 413,466 | 45.462 |
| rs174592 | G | A | 0.072 | 0.010 | 9.92E-14 | 413,466 | 55.098 |
| rs10791849 | A | T | -0.069 | 0.012 | 9.89E-09 | 413,466 | 32.892 |
| rs12575685 | A | G | 0.065 | 0.010 | 1.24E-10 | 413,466 | 41.673 |
| rs7108878 | G | T | 0.081 | 0.015 | 3.61E-08 | 413,466 | 30.365 |
| rs11062170 | C | G | 0.078 | 0.010 | 1.87E-15 | 413,466 | 63.181 |
| rs35306827 | A | G | -0.066 | 0.011 | 3.56E-09 | 413,466 | 34.621 |
| rs2693698 | G | A | 0.053 | 0.009 | 1.96E-08 | 413,466 | 31.790 |
| rs35958438 | A | G | -0.064 | 0.012 | 3.83E-08 | 413,466 | 30.113 |
| rs4447398 | C | A | -0.082 | 0.014 | 2.61E-09 | 413,466 | 35.478 |
| rs748455 | C | T | -0.067 | 0.010 | 5.01E-11 | 413,466 | 42.947 |
| rs28455634 | A | G | -0.063 | 0.010 | 2.63E-10 | 413,466 | 40.235 |
| rs7201930 | C | T | 0.059 | 0.010 | 1.89E-08 | 413,466 | 31.753 |
| rs4790841 | T | C | 0.073 | 0.013 | 3.14E-08 | 413,466 | 30.504 |
| rs61554907 | T | G | 0.087 | 0.015 | 1.64E-08 | 413,466 | 31.769 |
| rs228768 | T | G | -0.064 | 0.010 | 2.83E-10 | 413,466 | 39.864 |
| rs6104027 | G | A | -0.060 | 0.010 | 1.93E-10 | 413,466 | 40.295 |
| rs237460 | T | C | 0.055 | 0.009 | 4.25E-09 | 413,466 | 34.611 |
| rs13044225 | G | A | 0.055 | 0.010 | 8.50E-09 | 413,466 | 33.152 |
| rs5758064 | C | T | -0.052 | 0.009 | 2.01E-08 | 413,466 | 31.750 |

**Table S3. 154 SNPs associated with schizophrenia.**

| SNP | Effect  allele | Other allele | Effect allele  frequency | Beta | Se | Pval | Sample  size | F |
| --- | --- | --- | --- | --- | --- | --- | --- | --- |
| rs6673880 | G | A | 0.495 | 0.062 | 0.009 | 7.19E-12 | 127,906 | 46.871 |
| rs3795310 | T | C | 0.463 | -0.051 | 0.009 | 5.75E-09 | 127,906 | 34.360 |
| rs56335113 | G | A | 0.697 | -0.065 | 0.009 | 6.02E-12 | 127,906 | 47.377 |
| rs4653164 | T | C | 0.668 | 0.051 | 0.009 | 3.08E-08 | 127,906 | 30.855 |
| rs11210892 | A | G | 0.67 | -0.064 | 0.009 | 2.68E-12 | 127,906 | 48.694 |
| rs1892346 | A | T | 0.564 | 0.048 | 0.009 | 3.56E-08 | 127,906 | 30.253 |
| rs12129573 | A | C | 0.366 | 0.078 | 0.009 | 2.28E-18 | 127,906 | 76.414 |
| rs11165867 | T | C | 0.16 | 0.074 | 0.012 | 1.30E-10 | 127,906 | 41.030 |
| rs1198588 | T | A | 0.788 | 0.103 | 0.011 | 1.73E-21 | 127,906 | 90.246 |
| rs12138231 | A | T | 0.817 | 0.067 | 0.012 | 7.99E-09 | 127,906 | 33.356 |
| rs16851048 | C | T | 0.195 | 0.074 | 0.011 | 4.15E-12 | 127,906 | 48.475 |
| rs7515363 | T | C | 0.619 | -0.054 | 0.009 | 1.84E-09 | 127,906 | 36.139 |
| rs11587347 | G | C | 0.094 | 0.104 | 0.015 | 1.53E-12 | 127,906 | 49.952 |
| rs145071536 | C | T | 0.192 | 0.085 | 0.012 | 1.62E-12 | 127,906 | 50.292 |
| rs6715366 | A | G | 0.267 | 0.054 | 0.010 | 2.49E-08 | 127,906 | 31.103 |
| rs12712510 | C | T | 0.527 | -0.057 | 0.009 | 5.14E-11 | 127,906 | 43.531 |
| rs3770754 | G | C | 0.368 | -0.053 | 0.009 | 5.35E-09 | 127,906 | 33.788 |
| rs13011472 | G | C | 0.484 | 0.070 | 0.009 | 4.28E-16 | 127,906 | 65.482 |
| rs6546857 | G | A | 0.232 | 0.060 | 0.010 | 2.74E-09 | 127,906 | 35.062 |
| rs6721531 | T | A | 0.341 | -0.052 | 0.009 | 1.47E-08 | 127,906 | 32.278 |
| rs7575796 | G | A | 0.094 | -0.096 | 0.017 | 2.06E-08 | 127,906 | 31.347 |
| rs13016542 | C | T | 0.136 | -0.088 | 0.013 | 8.28E-12 | 127,906 | 46.858 |
| rs1881046 | T | G | 0.34 | -0.051 | 0.009 | 3.39E-08 | 127,906 | 30.373 |
| rs2909457 | A | G | 0.564 | -0.049 | 0.009 | 1.48E-08 | 127,906 | 31.721 |
| rs62183855 | C | A | 0.192 | -0.066 | 0.011 | 2.66E-09 | 127,906 | 35.458 |
| rs11693094 | T | C | 0.461 | -0.054 | 0.009 | 4.29E-10 | 127,906 | 39.103 |
| rs12151767 | A | G | 0.487 | -0.061 | 0.009 | 1.31E-12 | 127,906 | 50.484 |
| rs1451488 | G | A | 0.555 | 0.071 | 0.009 | 4.47E-16 | 127,906 | 66.403 |
| rs3739118 | A | G | 0.291 | -0.057 | 0.010 | 2.36E-09 | 127,906 | 36.005 |
| rs3791710 | C | T | 0.203 | -0.060 | 0.011 | 3.02E-08 | 127,906 | 30.868 |
| rs778371 | G | A | 0.286 | 0.081 | 0.010 | 1.49E-17 | 127,906 | 71.987 |
| rs17194490 | T | G | 0.161 | 0.078 | 0.012 | 1.80E-11 | 127,906 | 45.445 |
| rs12489270 | C | T | 0.375 | 0.058 | 0.009 | 7.47E-11 | 127,906 | 42.330 |
| rs1430894 | T | C | 0.483 | 0.053 | 0.009 | 6.15E-10 | 127,906 | 38.404 |
| rs6549963 | C | T | 0.416 | -0.048 | 0.009 | 4.31E-08 | 127,906 | 30.130 |
| rs9876421 | T | C | 0.343 | 0.063 | 0.009 | 9.19E-12 | 127,906 | 46.156 |
| rs2710323 | C | T | 0.49 | -0.078 | 0.009 | 1.23E-19 | 127,906 | 83.116 |
| rs6798742 | G | A | 0.307 | 0.061 | 0.009 | 4.57E-11 | 127,906 | 43.162 |
| rs60135207 | T | G | 0.415 | -0.050 | 0.009 | 1.53E-08 | 127,906 | 31.768 |
| rs167924 | G | A | 0.631 | 0.050 | 0.009 | 2.34E-08 | 127,906 | 31.111 |
| rs1604060 | G | A | 0.885 | 0.077 | 0.014 | 3.24E-08 | 127,906 | 30.411 |
| rs7634476 | G | A | 0.586 | 0.058 | 0.009 | 5.46E-11 | 127,906 | 42.997 |
| rs308697 | A | C | 0.436 | -0.050 | 0.009 | 8.83E-09 | 127,906 | 33.166 |
| rs7647398 | T | C | 0.2 | -0.077 | 0.011 | 1.07E-12 | 127,906 | 50.551 |
| rs35734242 | C | T | 0.426 | 0.051 | 0.009 | 1.37E-08 | 127,906 | 32.457 |
| rs215412 | A | G | 0.328 | 0.058 | 0.009 | 2.69E-10 | 127,906 | 40.209 |
| rs11941714 | A | G | 0.336 | -0.052 | 0.009 | 3.07E-08 | 127,906 | 30.779 |
| rs13107325 | T | C | 0.067 | 0.159 | 0.017 | 2.90E-21 | 127,906 | 89.238 |
| rs1427633 | C | G | 0.593 | -0.048 | 0.009 | 4.10E-08 | 127,906 | 30.130 |
| rs2333321 | G | A | 0.792 | -0.071 | 0.011 | 1.25E-11 | 127,906 | 45.986 |
| rs10035564 | G | A | 0.331 | 0.067 | 0.009 | 4.38E-13 | 127,906 | 52.724 |
| rs4700418 | G | C | 0.493 | 0.070 | 0.009 | 5.37E-16 | 127,906 | 65.103 |
| rs16867571 | G | A | 0.235 | -0.066 | 0.010 | 2.68E-10 | 127,906 | 39.913 |
| rs1901512 | C | T | 0.699 | -0.058 | 0.009 | 5.72E-10 | 127,906 | 38.600 |
| rs187557 | T | C | 0.848 | -0.067 | 0.012 | 2.03E-08 | 127,906 | 31.412 |
| rs10117 | A | G | 0.398 | -0.055 | 0.009 | 4.66E-10 | 127,906 | 39.062 |
| rs9687282 | G | T | 0.334 | 0.053 | 0.009 | 7.33E-09 | 127,906 | 33.410 |
| rs72802868 | T | G | 0.294 | -0.069 | 0.010 | 4.55E-13 | 127,906 | 51.959 |
| rs11740474 | T | A | 0.415 | 0.054 | 0.009 | 1.13E-09 | 127,906 | 37.232 |
| rs12652777 | C | T | 0.522 | -0.049 | 0.009 | 1.52E-08 | 127,906 | 32.199 |
| rs13195636 | C | A | 0.091 | -0.211 | 0.016 | 6.55E-40 | 127,906 | 175.278 |
| rs9461916 | C | T | 0.594 | 0.053 | 0.009 | 1.64E-09 | 127,906 | 36.679 |
| rs2815731 | A | C | 0.358 | -0.060 | 0.009 | 4.39E-11 | 127,906 | 43.478 |
| rs217336 | A | C | 0.434 | -0.050 | 0.009 | 8.05E-09 | 127,906 | 33.431 |
| rs634940 | T | G | 0.252 | 0.066 | 0.010 | 1.78E-11 | 127,906 | 44.980 |
| rs58120505 | C | T | 0.418 | -0.090 | 0.009 | 2.24E-24 | 127,906 | 103.676 |
| rs79210963 | C | T | 0.108 | 0.086 | 0.014 | 4.14E-10 | 127,906 | 39.041 |
| rs1914399 | G | C | 0.521 | -0.049 | 0.009 | 1.40E-08 | 127,906 | 31.857 |
| rs6943762 | C | T | 0.13 | -0.105 | 0.013 | 1.57E-15 | 127,906 | 63.393 |
| rs13233308 | T | C | 0.484 | -0.049 | 0.009 | 1.75E-08 | 127,906 | 32.073 |
| rs2252074 | G | T | 0.395 | 0.069 | 0.009 | 6.19E-15 | 127,906 | 60.599 |
| rs6974218 | C | A | 0.382 | -0.055 | 0.009 | 6.80E-10 | 127,906 | 38.044 |
| rs11534045 | A | G | 0.325 | -0.063 | 0.009 | 1.40E-11 | 127,906 | 45.594 |
| rs1593304 | G | A | 0.801 | 0.064 | 0.011 | 7.45E-09 | 127,906 | 33.349 |
| rs7798283 | G | T | 0.133 | -0.074 | 0.013 | 3.49E-08 | 127,906 | 30.499 |
| rs728055 | A | T | 0.358 | -0.067 | 0.009 | 8.85E-14 | 127,906 | 56.078 |
| rs10503253 | A | C | 0.206 | 0.062 | 0.011 | 4.53E-09 | 127,906 | 34.215 |
| rs4921741 | G | A | 0.261 | 0.056 | 0.010 | 1.21E-08 | 127,906 | 32.652 |
| rs73229090 | A | C | 0.112 | -0.103 | 0.014 | 4.34E-13 | 127,906 | 52.208 |
| rs79445414 | C | T | 0.04 | 0.123 | 0.022 | 2.80E-08 | 127,906 | 30.898 |
| rs6984242 | A | G | 0.6 | -0.055 | 0.009 | 3.85E-10 | 127,906 | 39.526 |
| rs10957321 | A | G | 0.511 | 0.048 | 0.009 | 3.48E-08 | 127,906 | 30.628 |
| rs1915019 | G | A | 0.75 | -0.057 | 0.010 | 6.57E-09 | 127,906 | 33.947 |
| rs10086619 | G | A | 0.161 | 0.072 | 0.012 | 4.97E-10 | 127,906 | 38.745 |
| rs4129585 | C | A | 0.562 | -0.075 | 0.009 | 5.11E-18 | 127,906 | 74.309 |
| rs11136325 | A | G | 0.579 | -0.054 | 0.009 | 3.05E-09 | 127,906 | 34.948 |
| rs498591 | T | A | 0.146 | 0.072 | 0.012 | 2.11E-09 | 127,906 | 35.896 |
| rs505061 | A | C | 0.488 | 0.053 | 0.009 | 5.80E-10 | 127,906 | 38.694 |
| rs2381411 | C | T | 0.402 | 0.050 | 0.009 | 1.25E-08 | 127,906 | 32.800 |
| rs500102 | C | T | 0.591 | -0.052 | 0.009 | 4.87E-09 | 127,906 | 34.516 |
| rs3824451 | C | T | 0.153 | 0.066 | 0.012 | 2.54E-08 | 127,906 | 30.901 |
| rs2078266 | G | A | 0.829 | -0.070 | 0.013 | 2.94E-08 | 127,906 | 30.513 |
| rs17731 | A | G | 0.365 | 0.052 | 0.009 | 4.37E-09 | 127,906 | 34.663 |
| rs6482437 | C | A | 0.888 | 0.099 | 0.014 | 3.33E-12 | 127,906 | 48.512 |
| rs12771371 | A | G | 0.311 | -0.052 | 0.009 | 1.94E-08 | 127,906 | 31.750 |
| rs61857878 | T | A | 0.254 | -0.060 | 0.010 | 4.44E-09 | 127,906 | 34.715 |
| rs11191580 | C | T | 0.09 | -0.132 | 0.016 | 1.77E-17 | 127,906 | 72.198 |
| rs11027839 | C | A | 0.5 | 0.052 | 0.009 | 2.40E-09 | 127,906 | 35.866 |
| rs4636654 | A | G | 0.402 | -0.048 | 0.009 | 4.89E-08 | 127,906 | 29.457 |
| rs12285419 | A | C | 0.188 | 0.085 | 0.011 | 1.05E-14 | 127,906 | 59.577 |
| rs708228 | T | C | 0.329 | 0.053 | 0.009 | 6.56E-09 | 127,906 | 33.665 |
| rs72943392 | C | G | 0.289 | 0.053 | 0.010 | 2.39E-08 | 127,906 | 31.052 |
| rs2514218 | T | C | 0.344 | -0.070 | 0.009 | 1.35E-14 | 127,906 | 58.715 |
| rs12293670 | G | A | 0.34 | -0.070 | 0.009 | 1.56E-14 | 127,906 | 58.715 |
| rs7112616 | C | T | 0.498 | -0.052 | 0.009 | 1.52E-09 | 127,906 | 36.847 |
| rs3802924 | C | A | 0.212 | -0.074 | 0.011 | 9.58E-12 | 127,906 | 46.446 |
| rs11223774 | G | A | 0.706 | -0.052 | 0.009 | 2.74E-08 | 127,906 | 31.191 |
| rs1860002 | T | C | 0.543 | -0.084 | 0.009 | 1.04E-21 | 127,906 | 92.776 |
| rs10876446 | C | G | 0.312 | 0.054 | 0.009 | 1.03E-08 | 127,906 | 33.004 |
| rs61937595 | T | C | 0.092 | -0.130 | 0.016 | 1.15E-15 | 127,906 | 64.493 |
| rs12303743 | C | G | 0.094 | 0.087 | 0.015 | 1.59E-09 | 127,906 | 36.414 |
| rs6538539 | T | G | 0.549 | -0.057 | 0.009 | 4.43E-11 | 127,906 | 43.615 |
| rs10861176 | A | G | 0.737 | 0.056 | 0.010 | 1.59E-08 | 127,906 | 32.075 |
| rs4766428 | T | C | 0.441 | 0.075 | 0.009 | 3.93E-17 | 127,906 | 71.021 |
| rs1615350 | T | C | 0.744 | -0.074 | 0.010 | 4.92E-14 | 127,906 | 56.409 |
| rs12833624 | T | C | 0.342 | 0.050 | 0.009 | 2.77E-08 | 127,906 | 31.111 |
| rs2455415 | T | C | 0.413 | 0.049 | 0.009 | 1.69E-08 | 127,906 | 31.634 |
| rs12877581 | C | G | 0.277 | 0.060 | 0.010 | 1.80E-09 | 127,906 | 36.245 |
| rs9318627 | C | A | 0.4 | -0.061 | 0.009 | 4.35E-12 | 127,906 | 48.363 |
| rs1953205 | A | T | 0.485 | 0.050 | 0.009 | 2.21E-08 | 127,906 | 31.442 |
| rs12883788 | T | C | 0.458 | 0.061 | 0.009 | 1.86E-12 | 127,906 | 49.648 |
| rs2999392 | T | C | 0.691 | 0.052 | 0.009 | 3.05E-08 | 127,906 | 30.366 |
| rs2332700 | G | C | 0.753 | -0.075 | 0.010 | 3.88E-14 | 127,906 | 57.542 |
| rs1540840 | C | G | 0.475 | -0.056 | 0.009 | 2.21E-09 | 127,906 | 35.871 |
| rs10873538 | G | T | 0.334 | 0.067 | 0.009 | 3.01E-13 | 127,906 | 53.407 |
| rs56205728 | A | G | 0.287 | 0.063 | 0.010 | 1.01E-10 | 127,906 | 42.188 |
| rs62018952 | C | T | 0.726 | 0.058 | 0.010 | 1.94E-09 | 127,906 | 36.251 |
| rs35351411 | C | A | 0.544 | 0.064 | 0.009 | 2.21E-13 | 127,906 | 53.281 |
| rs2456020 | T | C | 0.241 | -0.082 | 0.010 | 1.13E-15 | 127,906 | 63.997 |
| rs4779050 | G | T | 0.633 | -0.058 | 0.009 | 7.27E-11 | 127,906 | 42.463 |
| rs4702 | A | G | 0.56 | -0.084 | 0.009 | 2.79E-21 | 127,906 | 89.726 |
| rs8055219 | A | G | 0.229 | 0.067 | 0.010 | 5.69E-11 | 127,906 | 43.355 |
| rs3814883 | T | C | 0.467 | -0.067 | 0.009 | 1.58E-14 | 127,906 | 59.481 |
| rs149165 | G | T | 0.446 | -0.048 | 0.009 | 3.00E-08 | 127,906 | 30.694 |
| rs4575535 | G | A | 0.704 | 0.056 | 0.010 | 5.77E-09 | 127,906 | 33.783 |
| rs73292401 | A | T | 0.192 | 0.068 | 0.011 | 5.48E-10 | 127,906 | 38.468 |
| rs57433322 | G | C | 0.122 | -0.083 | 0.014 | 1.99E-09 | 127,906 | 35.741 |
| rs2696466 | G | A | 0.403 | -0.061 | 0.009 | 2.64E-11 | 127,906 | 44.249 |
| rs9304548 | A | C | 0.748 | -0.057 | 0.010 | 1.59E-08 | 127,906 | 32.151 |
| rs4632195 | T | C | 0.52 | 0.047 | 0.009 | 4.59E-08 | 127,906 | 30.118 |
| rs9636107 | G | A | 0.471 | 0.070 | 0.009 | 5.11E-16 | 127,906 | 66.057 |
| rs11664298 | A | G | 0.193 | 0.077 | 0.011 | 8.94E-13 | 127,906 | 51.360 |
| rs76838079 | T | C | 0.146 | 0.078 | 0.014 | 1.53E-08 | 127,906 | 31.951 |
| rs72986630 | T | C | 0.068 | 0.112 | 0.018 | 3.59E-10 | 127,906 | 39.357 |
| rs1000237 | A | T | 0.355 | 0.073 | 0.009 | 2.80E-16 | 127,906 | 67.656 |
| rs2053079 | G | A | 0.237 | 0.060 | 0.010 | 3.01E-09 | 127,906 | 35.171 |
| rs7251 | G | C | 0.328 | -0.064 | 0.009 | 8.29E-12 | 127,906 | 46.502 |
| rs4812325 | A | G | 0.616 | 0.072 | 0.009 | 8.96E-16 | 127,906 | 65.272 |
| rs11696755 | C | T | 0.184 | 0.064 | 0.011 | 7.26E-09 | 127,906 | 33.531 |
| rs113264400 | C | T | 0.048 | 0.112 | 0.020 | 2.86E-08 | 127,906 | 30.905 |
| rs132582 | T | C | 0.545 | -0.051 | 0.009 | 3.26E-09 | 127,906 | 35.164 |
| rs5751191 | C | T | 0.501 | 0.066 | 0.009 | 3.00E-14 | 127,906 | 58.176 |
| rs8138941 | A | G | 0.204 | 0.058 | 0.011 | 4.46E-08 | 127,906 | 30.038 |
| rs6010045 | C | T | 0.698 | 0.055 | 0.010 | 7.44E-09 | 127,906 | 33.396 |

**Table S4. 85 SNPs associated with cardiac arrhythmias.**

| **SNP** | **Effect  allele** | **Other allele** | **Effect allele  frequency** | **Beta** | **Se** | **Pval** | **Sample  size** | **F** |
| --- | --- | --- | --- | --- | --- | --- | --- | --- |
| rs1106270 | G | A | 0.323414 | 0.042 | 0.007 | 1.65E-10 | 381142 | 40.840 |
| rs188203 | T | C | 0.124139 | 0.052 | 0.009 | 3.54E-08 | 381142 | 30.386 |
| rs4654773 | G | A | 0.784944 | 0.043 | 0.008 | 9.87E-09 | 381142 | 32.866 |
| rs2885697 | T | G | 0.661009 | -0.038 | 0.007 | 4.75E-09 | 381142 | 34.290 |
| rs74738164 | A | G | 0.0217623 | 0.164 | 0.021 | 3.36E-15 | 381142 | 62.043 |
| rs12145374 | C | A | 0.166651 | -0.053 | 0.008 | 2.92E-10 | 381142 | 39.729 |
| rs75185665 | T | C | 0.0582419 | -0.089 | 0.013 | 2.56E-11 | 381142 | 44.485 |
| rs2335249 | C | T | 0.564915 | 0.046 | 0.006 | 2.79E-13 | 381142 | 53.349 |
| rs34515871 | T | C | 0.297483 | 0.081 | 0.007 | 3.00E-33 | 381142 | 144.333 |
| rs488488 | A | C | 0.962997 | -0.105 | 0.016 | 1.02E-10 | 381142 | 41.782 |
| rs651386 | T | A | 0.379802 | -0.047 | 0.006 | 2.28E-13 | 381142 | 53.748 |
| rs3737883 | G | A | 0.643547 | -0.047 | 0.006 | 2.15E-13 | 381142 | 53.866 |
| rs12986445 | A | T | 0.644713 | 0.037 | 0.006 | 1.53E-08 | 381142 | 32.016 |
| rs2723065 | G | A | 0.341832 | -0.051 | 0.007 | 1.25E-14 | 381142 | 59.456 |
| rs7574892 | A | G | 0.484181 | 0.042 | 0.006 | 2.08E-11 | 381142 | 44.890 |
| rs2288327 | G | A | 0.16787 | 0.073 | 0.008 | 8.25E-19 | 381142 | 78.439 |
| rs296790 | G | C | 0.522047 | -0.044 | 0.006 | 9.78E-13 | 381142 | 50.888 |
| rs7650482 | G | A | 0.609181 | 0.057 | 0.006 | 1.89E-19 | 381142 | 81.355 |
| rs12053903 | C | T | 0.406355 | -0.066 | 0.006 | 9.57E-26 | 381142 | 110.047 |
| rs7373065 | C | T | 0.97489 | -0.147 | 0.020 | 5.84E-14 | 381142 | 56.424 |
| rs34811474 | A | G | 0.229838 | -0.042 | 0.007 | 1.38E-08 | 381142 | 32.217 |
| rs17234610 | A | G | 0.0371771 | 0.120 | 0.016 | 2.83E-13 | 381142 | 53.323 |
| rs11931318 | A | G | 0.510068 | -0.037 | 0.006 | 1.62E-09 | 381142 | 36.384 |
| rs13121747 | A | G | 0.291015 | -0.099 | 0.007 | 1.37E-46 | 381142 | 205.421 |
| rs10026140 | G | T | 0.142916 | 0.262 | 0.009 | 1.00E-200 | 381142 | 917.772 |
| rs6838973 | T | C | 0.500003 | -0.107 | 0.006 | 2.85E-67 | 381142 | 300.286 |
| rs78049276 | C | A | 0.133938 | 0.050 | 0.009 | 4.77E-08 | 381142 | 29.808 |
| rs11725517 | C | T | 0.531298 | 0.038 | 0.006 | 7.84E-10 | 381142 | 37.801 |
| rs28424064 | C | A | 0.458915 | -0.034 | 0.006 | 4.82E-08 | 381142 | 29.787 |
| rs678897 | C | G | 0.2869 | 0.068 | 0.007 | 4.16E-23 | 381142 | 98.013 |
| rs3776299 | A | G | 0.505451 | 0.039 | 0.006 | 2.59E-10 | 381142 | 39.959 |
| rs10515522 | C | T | 0.192527 | 0.062 | 0.008 | 2.86E-15 | 381142 | 62.363 |
| rs56281418 | C | T | 0.413379 | -0.040 | 0.006 | 2.45E-10 | 381142 | 40.070 |
| rs113755256 | A | G | 0.104273 | -0.072 | 0.010 | 2.05E-12 | 381142 | 49.432 |
| rs9371048 | G | A | 0.308 | 0.041 | 0.007 | 8.29E-10 | 381142 | 37.691 |
| rs6456496 | C | G | 0.256352 | 0.056 | 0.007 | 3.12E-15 | 381142 | 62.188 |
| rs436388 | C | T | 0.5074 | 0.047 | 0.006 | 6.29E-14 | 381142 | 56.279 |
| rs3176326 | A | G | 0.173361 | -0.070 | 0.008 | 3.16E-17 | 381142 | 71.243 |
| rs608815 | T | G | 0.196917 | 0.049 | 0.008 | 4.26E-10 | 381142 | 38.991 |
| rs1402538 | A | G | 0.332534 | -0.045 | 0.007 | 6.01E-12 | 381142 | 47.326 |
| rs10455872 | G | A | 0.045772 | 0.091 | 0.015 | 7.05E-10 | 381142 | 38.006 |
| rs61707771 | C | T | 0.116931 | 0.058 | 0.010 | 1.79E-09 | 381142 | 36.189 |
| rs202142 | C | T | 0.343629 | 0.039 | 0.007 | 2.89E-09 | 381142 | 35.257 |
| rs11773845 | A | C | 0.561745 | 0.079 | 0.006 | 1.27E-36 | 381142 | 159.763 |
| rs60425056 | T | C | 0.0965549 | 0.082 | 0.010 | 4.49E-15 | 381142 | 61.474 |
| rs7789146 | A | G | 0.229082 | -0.056 | 0.007 | 4.17E-14 | 381142 | 57.087 |
| rs28583947 | G | C | 0.0852889 | -0.066 | 0.011 | 4.63E-09 | 381142 | 34.339 |
| rs17430357 | T | A | 0.16124 | 0.047 | 0.008 | 2.23E-08 | 381142 | 31.288 |
| rs12334478 | G | C | 0.423248 | -0.037 | 0.006 | 4.57E-09 | 381142 | 34.367 |
| rs7853096 | T | C | 0.0993398 | 0.064 | 0.010 | 6.16E-10 | 381142 | 38.270 |
| rs4977575 | G | C | 0.429315 | 0.045 | 0.006 | 5.18E-13 | 381142 | 52.135 |
| rs1020963 | G | A | 0.878015 | 0.056 | 0.010 | 5.19E-09 | 381142 | 34.117 |
| rs635634 | C | T | 0.799089 | -0.049 | 0.008 | 2.91E-10 | 381142 | 39.736 |
| rs4240499 | C | T | 0.377358 | -0.040 | 0.006 | 5.62E-10 | 381142 | 38.450 |
| rs11525236 | T | G | 0.212752 | 0.046 | 0.008 | 1.41E-09 | 381142 | 36.658 |
| rs76443711 | C | G | 0.146703 | -0.072 | 0.009 | 4.58E-16 | 381142 | 65.971 |
| rs72816711 | C | T | 0.107668 | 0.060 | 0.010 | 1.85E-09 | 381142 | 36.129 |
| rs7067666 | T | C | 0.452599 | 0.042 | 0.006 | 1.30E-11 | 381142 | 45.813 |
| rs728713 | G | A | 0.919769 | -0.075 | 0.011 | 3.61E-11 | 381142 | 43.814 |
| rs10769602 | T | A | 0.214709 | 0.045 | 0.008 | 5.37E-09 | 381142 | 34.050 |
| rs75557443 | T | C | 0.0982091 | 0.081 | 0.010 | 6.90E-15 | 381142 | 60.626 |
| rs7298923 | T | A | 0.820837 | 0.050 | 0.008 | 7.21E-10 | 381142 | 37.962 |
| rs883079 | T | C | 0.687118 | 0.049 | 0.007 | 1.93E-13 | 381142 | 54.075 |
| rs6488930 | G | A | 0.289421 | 0.039 | 0.007 | 1.08E-08 | 381142 | 32.694 |
| rs2315545 | T | C | 0.402469 | 0.039 | 0.006 | 8.96E-10 | 381142 | 37.540 |
| rs4981979 | T | C | 0.745811 | 0.040 | 0.007 | 2.35E-08 | 381142 | 31.179 |
| rs1152589 | T | A | 0.522491 | -0.038 | 0.006 | 7.54E-10 | 381142 | 37.877 |
| rs17810431 | A | T | 0.574864 | -0.040 | 0.006 | 1.84E-10 | 381142 | 40.627 |
| rs11858506 | T | C | 0.618328 | -0.041 | 0.006 | 1.18E-10 | 381142 | 41.494 |
| rs74022964 | T | C | 0.206109 | 0.063 | 0.008 | 1.29E-16 | 381142 | 68.472 |
| rs150695505 | A | C | 0.0605521 | 0.096 | 0.013 | 9.08E-14 | 381142 | 55.557 |
| rs139277460 | G | C | 0.0133289 | 0.187 | 0.026 | 1.64E-12 | 381142 | 49.876 |
| rs9941349 | T | C | 0.409229 | 0.042 | 0.006 | 4.17E-11 | 381142 | 43.534 |
| rs12932445 | C | T | 0.231452 | 0.094 | 0.007 | 1.93E-37 | 381142 | 163.518 |
| rs148799368 | A | G | 0.0958639 | -0.058 | 0.011 | 3.84E-08 | 381142 | 30.228 |
| rs216200 | T | C | 0.652806 | -0.041 | 0.006 | 1.93E-10 | 381142 | 40.537 |
| rs145153053 | G | A | 0.197831 | 0.063 | 0.008 | 5.38E-16 | 381142 | 65.654 |
| rs6504403 | T | G | 0.829246 | -0.050 | 0.008 | 1.10E-09 | 381142 | 37.137 |
| rs167479 | G | T | 0.575959 | 0.045 | 0.006 | 1.30E-12 | 381142 | 50.336 |
| rs117831371 | T | C | 0.0218193 | 0.175 | 0.021 | 3.43E-17 | 381142 | 71.081 |
| rs2025811 | C | T | 0.808059 | -0.047 | 0.008 | 3.34E-09 | 381142 | 34.973 |
| rs78412528 | A | G | 0.167278 | 0.050 | 0.008 | 1.24E-09 | 381142 | 36.908 |
| rs2834618 | G | T | 0.115057 | -0.058 | 0.010 | 4.29E-09 | 381142 | 34.487 |
| rs62223042 | G | A | 0.517032 | -0.035 | 0.006 | 1.56E-08 | 381142 | 31.972 |
| rs464901 | C | T | 0.315638 | -0.042 | 0.007 | 4.94E-10 | 381142 | 38.700 |

**Table S5. 128 SNPs associated with atrial fibrillation and flutter.**

| **SNP** | **Effect  allele** | **Other allele** | **Effect allele  frequency** | **Beta** | **Se** | **Pval** | **Sample  size** | **F** |
| --- | --- | --- | --- | --- | --- | --- | --- | --- |
| rs880315 | C | T | 0.411539 | 0.077 | 0.009 | 2.12E-18 | 316342 | 76.575 |
| rs11576963 | A | T | 0.189277 | -0.070 | 0.011 | 2.61E-10 | 316342 | 39.944 |
| rs72690464 | G | T | 0.0234659 | 0.165 | 0.028 | 2.95E-09 | 316342 | 35.218 |
| rs7528118 | A | G | 0.257402 | 0.062 | 0.010 | 3.11E-10 | 316342 | 39.602 |
| rs12090194 | T | C | 0.276184 | -0.081 | 0.010 | 3.57E-17 | 316342 | 71.002 |
| rs72699046 | C | G | 0.154511 | 0.069 | 0.012 | 8.78E-09 | 316342 | 33.093 |
| rs78581286 | A | G | 0.0580485 | -0.151 | 0.019 | 6.23E-16 | 316342 | 65.362 |
| rs2335249 | C | T | 0.565714 | 0.074 | 0.009 | 2.59E-17 | 316342 | 71.632 |
| rs12754189 | C | T | 0.298907 | 0.142 | 0.009 | 2.37E-52 | 316342 | 231.837 |
| rs7367758 | T | C | 0.205451 | -0.065 | 0.011 | 1.42E-09 | 316342 | 36.643 |
| rs56147946 | T | C | 0.0372869 | 0.153 | 0.022 | 4.06E-12 | 316342 | 48.096 |
| rs651386 | T | A | 0.379018 | -0.080 | 0.009 | 3.57E-19 | 316342 | 80.096 |
| rs3737883 | G | A | 0.642881 | -0.079 | 0.009 | 1.20E-18 | 316342 | 77.694 |
| rs10158275 | T | G | 0.346131 | 0.053 | 0.009 | 3.96E-09 | 316342 | 34.645 |
| rs11126296 | C | T | 0.386587 | -0.052 | 0.009 | 5.60E-09 | 316342 | 33.969 |
| rs9309001 | T | G | 0.389707 | -0.048 | 0.009 | 4.30E-08 | 316342 | 30.008 |
| rs114962644 | G | A | 0.179253 | 0.067 | 0.011 | 3.04E-09 | 316342 | 35.160 |
| rs2723064 | C | T | 0.341207 | -0.065 | 0.009 | 1.11E-12 | 316342 | 50.635 |
| rs6546692 | A | T | 0.480987 | 0.051 | 0.009 | 2.45E-09 | 316342 | 35.578 |
| rs17490854 | C | A | 0.136419 | -0.075 | 0.013 | 2.53E-09 | 316342 | 35.513 |
| rs7574892 | A | G | 0.484844 | 0.055 | 0.009 | 1.60E-10 | 316342 | 40.898 |
| rs2288327 | G | A | 0.168218 | 0.120 | 0.011 | 3.19E-26 | 316342 | 112.227 |
| rs295141 | G | A | 0.369009 | -0.049 | 0.009 | 3.56E-08 | 316342 | 30.375 |
| rs4642101 | G | T | 0.609747 | 0.087 | 0.009 | 6.30E-23 | 316342 | 97.190 |
| rs12053903 | C | T | 0.405599 | -0.069 | 0.009 | 3.95E-15 | 316342 | 61.723 |
| rs9856387 | C | T | 0.620645 | -0.058 | 0.009 | 7.22E-11 | 316342 | 42.458 |
| rs1843050 | C | T | 0.261035 | -0.058 | 0.010 | 3.04E-09 | 316342 | 35.156 |
| rs7633500 | A | G | 0.444901 | -0.051 | 0.009 | 5.85E-09 | 316342 | 33.885 |
| rs9846313 | G | C | 0.647049 | 0.059 | 0.009 | 5.80E-11 | 316342 | 42.886 |
| rs28456085 | T | A | 0.423411 | -0.051 | 0.009 | 3.11E-09 | 316342 | 35.115 |
| rs12509595 | C | T | 0.311733 | 0.083 | 0.009 | 2.68E-19 | 316342 | 80.660 |
| rs2346029 | G | A | 0.102369 | 0.104 | 0.014 | 1.15E-13 | 316342 | 55.084 |
| rs11931318 | A | G | 0.509988 | -0.069 | 0.009 | 6.19E-16 | 316342 | 65.375 |
| rs28730909 | A | T | 0.312235 | 0.064 | 0.009 | 4.34E-12 | 316342 | 47.965 |
| rs243987 | T | G | 0.648939 | -0.067 | 0.009 | 7.24E-14 | 316342 | 56.003 |
| rs13121747 | A | G | 0.289192 | -0.159 | 0.010 | 1.85E-61 | 316342 | 273.621 |
| rs6854883 | T | C | 0.189212 | 0.335 | 0.011 | 1.00E-200 | 316342 | 989.549 |
| rs6838973 | T | C | 0.497848 | -0.179 | 0.009 | 2.48E-97 | 316342 | 438.347 |
| rs150939405 | C | T | 0.0337505 | 0.135 | 0.024 | 1.15E-08 | 316342 | 32.571 |
| rs1021956 | T | G | 0.857819 | -0.084 | 0.012 | 7.66E-12 | 316342 | 46.851 |
| rs55729900 | G | A | 0.153823 | -0.081 | 0.012 | 1.08E-11 | 316342 | 46.177 |
| rs6555042 | C | T | 0.491087 | -0.051 | 0.009 | 4.79E-09 | 316342 | 34.271 |
| rs12656497 | C | T | 0.585485 | 0.051 | 0.009 | 5.01E-09 | 316342 | 34.186 |
| rs6878512 | C | T | 0.802481 | 0.068 | 0.011 | 4.13E-10 | 316342 | 39.051 |
| rs1019457 | T | G | 0.279253 | -0.056 | 0.010 | 6.92E-09 | 316342 | 33.556 |
| rs338623 | G | A | 0.629999 | -0.057 | 0.009 | 1.29E-10 | 316342 | 41.324 |
| rs10039629 | T | G | 0.420728 | -0.053 | 0.009 | 1.04E-09 | 316342 | 37.251 |
| rs678897 | C | G | 0.287424 | 0.101 | 0.009 | 2.86E-26 | 316342 | 112.440 |
| rs370479 | G | A | 0.123863 | 0.076 | 0.013 | 6.93E-09 | 316342 | 33.555 |
| rs3776299 | A | G | 0.505261 | 0.058 | 0.009 | 2.64E-11 | 316342 | 44.425 |
| rs10515522 | C | T | 0.1933 | 0.070 | 0.011 | 1.02E-10 | 316342 | 41.777 |
| rs56388234 | A | G | 0.371147 | -0.053 | 0.009 | 3.41E-09 | 316342 | 34.932 |
| rs6884881 | C | T | 0.65385 | -0.054 | 0.009 | 2.81E-09 | 316342 | 35.312 |
| rs10054063 | T | A | 0.413547 | -0.062 | 0.009 | 2.13E-12 | 316342 | 49.359 |
| rs59430691 | A | G | 0.103923 | -0.104 | 0.014 | 2.67E-13 | 316342 | 53.441 |
| rs34969716 | A | G | 0.331606 | 0.074 | 0.009 | 7.40E-16 | 316342 | 65.022 |
| rs75746146 | G | C | 0.289039 | -0.066 | 0.010 | 4.67E-12 | 316342 | 47.821 |
| rs3176326 | A | G | 0.172365 | -0.072 | 0.011 | 3.36E-10 | 316342 | 39.456 |
| rs9341528 | T | C | 0.146369 | 0.076 | 0.012 | 2.81E-10 | 316342 | 39.803 |
| rs12196324 | A | G | 0.193913 | 0.061 | 0.011 | 2.60E-08 | 316342 | 30.987 |
| rs868155 | T | C | 0.291839 | -0.074 | 0.010 | 9.93E-15 | 316342 | 59.910 |
| rs117984853 | T | G | 0.130295 | 0.079 | 0.013 | 1.05E-09 | 316342 | 37.234 |
| rs55730499 | T | C | 0.0454781 | 0.132 | 0.021 | 1.27E-10 | 316342 | 41.357 |
| rs10232964 | A | G | 0.485999 | 0.065 | 0.009 | 3.01E-14 | 316342 | 57.726 |
| rs1725074 | T | C | 0.142862 | 0.073 | 0.012 | 3.20E-09 | 316342 | 35.058 |
| rs2023844 | A | G | 0.903768 | 0.125 | 0.015 | 4.82E-17 | 316342 | 70.411 |
| rs10276861 | A | G | 0.810133 | -0.061 | 0.011 | 2.68E-08 | 316342 | 30.924 |
| rs3807989 | G | A | 0.569767 | 0.102 | 0.009 | 9.74E-32 | 316342 | 137.423 |
| rs55985730 | G | T | 0.0817361 | 0.119 | 0.016 | 2.76E-14 | 316342 | 57.899 |
| rs3778873 | G | C | 0.225836 | -0.062 | 0.010 | 2.58E-09 | 316342 | 35.478 |
| rs17060681 | A | G | 0.0846352 | -0.092 | 0.016 | 4.50E-09 | 316342 | 34.395 |
| rs17477457 | C | T | 0.114664 | 0.074 | 0.013 | 3.67E-08 | 316342 | 30.316 |
| rs35719208 | T | C | 0.224491 | -0.070 | 0.010 | 1.58E-11 | 316342 | 45.429 |
| rs17430357 | T | A | 0.161678 | 0.072 | 0.012 | 5.58E-10 | 316342 | 38.465 |
| rs77325656 | G | A | 0.0690275 | 0.098 | 0.017 | 6.77E-09 | 316342 | 33.598 |
| rs10108814 | G | C | 0.412056 | -0.053 | 0.009 | 1.09E-09 | 316342 | 37.150 |
| rs1333048 | C | A | 0.418608 | 0.073 | 0.009 | 6.63E-17 | 316342 | 69.780 |
| rs1016013 | G | A | 0.652924 | -0.063 | 0.009 | 2.43E-12 | 316342 | 49.105 |
| rs579459 | T | C | 0.778123 | -0.068 | 0.010 | 5.13E-11 | 316342 | 43.125 |
| rs599087 | G | A | 0.113667 | 0.088 | 0.014 | 1.69E-10 | 316342 | 40.801 |
| rs4240499 | C | T | 0.377267 | -0.061 | 0.009 | 7.96E-12 | 316342 | 46.775 |
| rs11525236 | T | G | 0.213393 | 0.084 | 0.011 | 1.88E-15 | 316342 | 63.186 |
| rs76443711 | C | G | 0.146038 | -0.106 | 0.012 | 6.14E-18 | 316342 | 74.475 |
| rs143073999 | A | C | 0.0536545 | -0.115 | 0.020 | 3.61E-09 | 316342 | 34.823 |
| rs12415501 | T | C | 0.0873892 | 0.138 | 0.015 | 5.89E-20 | 316342 | 83.654 |
| rs35176054 | A | T | 0.135363 | 0.119 | 0.013 | 2.94E-21 | 316342 | 89.584 |
| rs3813879 | T | G | 0.694999 | 0.055 | 0.009 | 6.33E-09 | 316342 | 33.731 |
| rs75557443 | T | C | 0.0990738 | 0.131 | 0.014 | 5.67E-20 | 316342 | 83.732 |
| rs11045480 | C | T | 0.610664 | 0.049 | 0.009 | 3.62E-08 | 316342 | 30.343 |
| rs17287293 | G | A | 0.105488 | -0.091 | 0.014 | 1.05E-10 | 316342 | 41.730 |
| rs11048425 | G | C | 0.461216 | 0.052 | 0.009 | 1.64E-09 | 316342 | 36.365 |
| rs11170778 | A | G | 0.162699 | 0.071 | 0.012 | 2.50E-09 | 316342 | 35.541 |
| rs2958126 | G | T | 0.769211 | -0.060 | 0.010 | 4.12E-09 | 316342 | 34.565 |
| rs141719954 | C | G | 0.101708 | -0.084 | 0.014 | 5.78E-09 | 316342 | 33.908 |
| rs7135659 | A | G | 0.701871 | 0.108 | 0.009 | 1.84E-30 | 316342 | 131.588 |
| rs34362160 | C | A | 0.433303 | 0.063 | 0.009 | 4.96E-13 | 316342 | 52.222 |
| rs2229842 | T | C | 0.28443 | 0.065 | 0.010 | 1.00E-11 | 316342 | 46.328 |
| rs12855310 | T | A | 0.218897 | 0.067 | 0.010 | 1.54E-10 | 316342 | 40.977 |
| rs3729833 | T | C | 0.119612 | 0.076 | 0.013 | 8.49E-09 | 316342 | 33.158 |
| rs8010040 | C | A | 0.744423 | 0.057 | 0.010 | 1.18E-08 | 316342 | 32.519 |
| rs1266927 | A | C | 0.434111 | -0.068 | 0.009 | 5.00E-15 | 316342 | 61.260 |
| rs17810431 | A | T | 0.575004 | -0.069 | 0.009 | 2.21E-15 | 316342 | 62.867 |
| rs74022964 | T | C | 0.207315 | 0.102 | 0.011 | 5.15E-22 | 316342 | 93.029 |
| rs150695505 | A | C | 0.0608439 | 0.142 | 0.018 | 1.47E-15 | 316342 | 63.666 |
| rs2521501 | T | A | 0.265912 | 0.057 | 0.010 | 6.11E-09 | 316342 | 33.799 |
| rs139277460 | G | C | 0.0134688 | 0.286 | 0.036 | 3.39E-15 | 316342 | 62.024 |
| rs140185678 | A | G | 0.0451341 | 0.128 | 0.021 | 4.31E-10 | 316342 | 38.969 |
| rs186918006 | A | C | 0.279251 | 0.057 | 0.010 | 6.93E-09 | 316342 | 33.555 |
| rs11075985 | A | C | 0.426895 | 0.057 | 0.009 | 6.23E-11 | 316342 | 42.746 |
| rs76844366 | A | T | 0.108546 | 0.079 | 0.014 | 1.33E-08 | 316342 | 32.287 |
| rs72789392 | C | T | 0.0874861 | 0.098 | 0.015 | 1.16E-10 | 316342 | 41.535 |
| rs12932445 | C | T | 0.233058 | 0.175 | 0.010 | 1.91E-67 | 316342 | 301.082 |
| rs216193 | G | A | 0.631223 | -0.062 | 0.009 | 2.69E-12 | 316342 | 48.905 |
| rs6504672 | A | T | 0.175659 | 0.105 | 0.011 | 7.61E-21 | 316342 | 87.703 |
| rs6504403 | T | G | 0.829701 | -0.069 | 0.011 | 1.39E-09 | 316342 | 36.687 |
| rs312759 | G | A | 0.391351 | 0.052 | 0.009 | 2.71E-09 | 316342 | 35.382 |
| rs116913163 | G | A | 0.0443295 | 0.120 | 0.021 | 9.42E-09 | 316342 | 32.958 |
| rs9945890 | T | G | 0.632074 | 0.054 | 0.009 | 1.32E-09 | 316342 | 36.780 |
| rs740404 | A | G | 0.127404 | 0.085 | 0.013 | 5.88E-11 | 316342 | 42.860 |
| rs167479 | G | T | 0.574019 | 0.073 | 0.009 | 5.37E-17 | 316342 | 70.196 |
| rs11673196 | T | C | 0.206577 | 0.060 | 0.011 | 1.97E-08 | 316342 | 31.520 |
| rs117831371 | T | C | 0.0221044 | 0.290 | 0.028 | 1.29E-24 | 316342 | 104.897 |
| rs6054135 | T | C | 0.627349 | 0.054 | 0.009 | 1.95E-09 | 316342 | 36.023 |
| rs6113183 | T | C | 0.690574 | -0.062 | 0.009 | 1.93E-11 | 316342 | 45.045 |
| rs73306888 | A | G | 0.163535 | 0.093 | 0.012 | 1.43E-15 | 316342 | 63.720 |
| rs2834618 | G | T | 0.114447 | -0.082 | 0.014 | 2.05E-09 | 316342 | 35.922 |
| rs2836949 | A | C | 0.555586 | -0.059 | 0.009 | 1.49E-11 | 316342 | 45.552 |
| rs464901 | C | T | 0.315125 | -0.055 | 0.009 | 4.80E-09 | 316342 | 34.271 |

**Table S6. 6 SNPs associated with paroxysmal tachycardia.**

| **SNP** | **Effect  allele** | **Other allele** | **Effect allele  frequency** | **Beta** | **Se** | **Pval** | **Sample  size** | **F** |
| --- | --- | --- | --- | --- | --- | --- | --- | --- |
| rs6804918 | G | A | 0.40964 | -0.083 | 0.014 | 8.05E-10 | 265688 | 37.747 |
| rs13104866 | A | G | 0.449325 | -0.077 | 0.013 | 9.51E-09 | 265688 | 32.938 |
| rs6843082 | A | G | 0.694949 | -0.094 | 0.014 | 5.76E-11 | 265688 | 42.900 |
| rs77999046 | C | T | 0.159763 | 0.139 | 0.018 | 2.79E-15 | 265688 | 62.410 |
| rs34930763 | G | C | 0.167873 | 0.095 | 0.017 | 4.63E-08 | 265688 | 29.866 |
| rs1858810 | G | A | 0.423766 | -0.088 | 0.013 | 5.05E-11 | 265688 | 43.157 |

**Table S7. 8 SNPs associated with paroxysmal tachycardia.**

| **SNP** | **Effect  allele** | **Other allele** | **Effect allele  frequency** | **Beta** | **Se** | **Pval** | **Sample  size** | **F** |
| --- | --- | --- | --- | --- | --- | --- | --- | --- |
| rs72646874 | C | A | 0.0524251 | 0.216 | 0.035 | 8.31E-10 | 389193 | 37.686 |
| rs56005624 | G | T | 0.10074 | 0.175 | 0.027 | 4.25E-11 | 389193 | 43.494 |
| rs2932976 | A | G | 0.324712 | 0.143 | 0.018 | 3.42E-16 | 389193 | 66.546 |
| rs6854111 | T | A | 0.231103 | 0.112 | 0.020 | 1.56E-08 | 389193 | 31.971 |
| rs11957898 | C | T | 0.19791 | 0.132 | 0.021 | 1.67E-10 | 389193 | 40.820 |
| rs3132139 | G | A | 0.71198 | -0.124 | 0.018 | 1.20E-11 | 389193 | 45.965 |
| rs11764098 | G | C | 0.026825 | 0.306 | 0.048 | 2.42E-10 | 389193 | 40.096 |
| rs7095308 | A | G | 0.262606 | 0.108 | 0.019 | 7.60E-09 | 389193 | 33.376 |
